# Oral Janus kinase inhibitors in the treatment of atopic dermatitis: A systematic review and meta‐analysis

**DOI:** 10.1002/ski2.133

**Published:** 2022-05-23

**Authors:** Kevin P. Lee, John Plante, Jeffrey E. Korte, Dirk M. Elston

**Affiliations:** ^1^ University of Texas at Houston McGovern Medical School Houston Texas USA; ^2^ Department of Dermatology and Dermatologic Surgery Medical University of South Carolina Charleston South Carolina USA; ^3^ Department of Public Health Sciences Medical University of South Carolina Charleston South Carolina USA

## Abstract

**Background:**

Janus kinase (JAK) inhibitors are being evaluated as promising upcoming treatments for atopic dermatitis (AD).

**Objectives:**

To systematically assess the efficacy of oral JAK inhibitors in patients with AD and provide comparisons among JAK inhibitors.

**Methods:**

A systematic literature review of JAK inhibitors in the treatment of AD was conducted and reported based on Preferred Reporting Items for Systematic Reviews and Meta‐Analyses using PubMed, ClinicalTrials.gov, CENTRAL, MEDLINE/Ovid, Embase and sponsor websites from inception to 30 September 2021. References of relevant articles were reviewed by two authors. Only RCTs of JAK inhibitors for treating AD with more than one study were included. Data was extracted and the meta‐analysis was performed using the metan procedure in STATA version 12.1. Risk of bias was assessed with the Cochrane Risk of Bias Tool. The four outcomes analysed included Eczema Area Severity Index (EASI)‐75 response (≥75% improvement of EASI score from baseline), percent change in EASI score, percent of subjects achieving Investigator Global Assessment (IGA) of clear or almost clear (IGA 0/1), and ≥ 4‐point improvement in pruritus numerical rating scale (NRS).

**Results:**

Fourteen randomized controlled trials (7051 subjects) assessing three different oral JAK inhibitors (abrocitinib, baricitinib and upadacitinib) in patients with moderate‐to‐severe AD were included in the meta‐analysis. Abrocitinib (100 and 200 mg), baricitinib (1, 2 and 4 mg) and upadacitinib (15 and 30 mg) were all found to be more efficacious compared to placebo in all four outcomes analysed. Upadacitinib 30 mg was more effective than all other dosages of JAK inhibitors in achieving EASI‐75, decrease in percent change of EASI, IGA 0/1 response rate, and ≥ 4‐point improvement in pruritus NRS.

**Conclusions:**

JAK inhibitors were found to be an effective treatment for AD. Upadacitinib, at 30 mg, was found to be the most efficacious oral JAK inhibitor for AD. More clinical trial studies with comparisons among JAK inhibitors are needed to confirm these results as well as explore long‐term efficacy and safety of these molecules.

## Graphical abstract

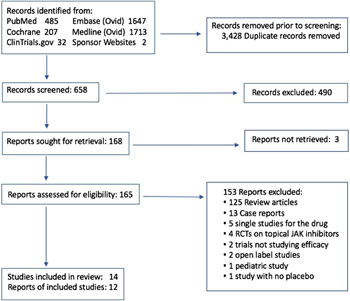

1

**Key points**

**What's already known about this topic?**

- Prior meta‐analyses have shown that Janus kinase (JAK) inhibitors are effective compared to placebo in treating atopic dermatitis.

**What does this study add?**

- Our study is the first meta‐analysis that looks at specific dosages of oral JAK inhibitors in atopic dermatitis to provide comparisons among JAK inhibitors.
- We found that upadacitinib, specifically at 30 mg, is the most efficacious oral JAK inhibitor for the treatment of atopic dermatitis.

## 2 INTRODUCTION

Atopic dermatitis (AD) is a common inflammatory skin disorder found in up to 20% of children and 10% of adults worldwide. This condition is characterised by eczematous lesions, intense pruritus and a chronic or relapsing disease course.^1^ Patients with AD not only suffer physically, but also psychologically and emotionally including embarrassment, anger and depression. Significant sleep disturbance may lead to decreased work production and lower school performance. Furthermore, the families of patients with AD suffer from increased stress and sleep deprivation.^2^, ^3^ The pathogenesis of AD is complex and includes barrier dysregulation, genetics, alteration in the skin microbiome, and a type‐2 predominant immune dysfunction.^1^ AD is driven by an increased T‐cell type 2 (Th2) response which releases cytokines such as IL‐4, IL‐5, IL‐13 and IL‐31.^4^

In addition to emollients, topical therapy is the mainstay of treatment for AD including corticosteroids, calcineurin inhibitors and phosphodiesterase inhibitors. In patients with severe AD or who are not controlled with topical therapy, phototherapy or systemic therapy such as corticosteroids, cyclosporine, methotrexate, or other immunosuppressive agents are recommended. Dupilumab, an IL‐4 and IL‐13 receptor inhibitor, was the first biologic approved for the treatment of AD. However, many patients did not achieve clear or almost clear skin and alternative treatment options are needed. A recent study comparing dupilumab and upadacitinib, a small molecule JAK inhibitor, in adults with moderate‐to‐severe AD found that upadacitinib was more superior in efficacy.^5^ Evidence for the Janus kinase‐signal transducer and activator of transcription (JAK‐STAT) pathway in treating AD has become more robust in recent years. The JAK‐STAT pathway consists of four Janus kinases [JAK1, JAK2, JAK3, tyrosine kinase 2 (TYK2)] and seven STAT proteins (STAT1, STAT2, STAT3, STAT4, STAT5A, STAT5B and STAT6). Janus kinases (JAKs) reside in the cytoplasm and bind to type 1 and type 2 cytokine receptors. Upon activation by the specific ligand binding, JAKs are phosphorylated. Subsequently, the JAKs phosphorylate their associated STAT protein, which dimerises and migrates to the nucleus, thereby regulating gene transcription.^6^ Numerous cytokines have been identified to stimulate the JAK‐STAT pathway including cytokines associated with AD (IL‐4, IL‐5 and IL‐13).^7^

JAK inhibitors are small molecules that diffuse into cells and inhibit the kinase portion of JAKs. Phosphorylation is blocked and transduction of intracellular signalling is inhibited. JAK inhibitors are formulated as oral or topical agents and can be grouped into first or second generation drugs. The first generation of JAK inhibitors were less selective in their target binding than the second generation.^8^ Many first generation JAK inhibitors include JAK2 as a target. JAK2 controls erythropoietin, thrombopoietin, IL‐11, G‐CSF and GM‐CSF signalling. Inhibition of JAK2 can lead to cytopenias including anaemia and neutropenia.^9^ The first generation of JAK inhibitors consists of tofacitinib, ruxolitinib, baricitinib, delgocitinib and oclacitinib. Oclacitinib is currently only approved for veterinary use.^10^ Multiple studies are currently underway to investigate both first and second generation JAK inhibitors for AD therapy. In our study, we performed a systematic review and meta‐analysis of randomized controlled trials (RCT) to assess the evidence for JAK inhibitor use in the treatment of AD.

## 3 METHODS

### 3.1 Data source and literature search

This study was conducted based on Preferred Reporting Items for Systematic Reviews and Meta‐Analyses (PRISMA) recommendations.^11^ A systematic literature review in the use of JAK inhibitors for the treatment of AD was conducted using PubMed, Cochrane Central Register of Controlled Trials (CENTRAL), Ovid MEDLINE, Embase (Ovid), ClinicalTrials.gov, and sponsor websites from the earliest publication date to 30 September 2021. References of relevant articles were also reviewed. The keywords ‘AD’ and ‘JAK inhibitor’ or the compound or generic name of JAK inhibitors (tofacitinib, baricitinib, oclacitinib, upadacitinib, itacitinib, momelotinib, peficitinib, decernotinib, fedratinib, pacritinib, filgotinib, gandotinib, solcitinib, lestaurtinib, PF‐06651600, BMS 986165, PF‐06700841, abrocitinib, gusacitinib, cerdulatinib, SHR0302, ATI‐50001, ATI‐50002, ATI‐1777, PF 06826647, CTP‐543, ATI‐501) were used for the search.

### 3.2 Eligibility criteria

**FIGURE 1:**
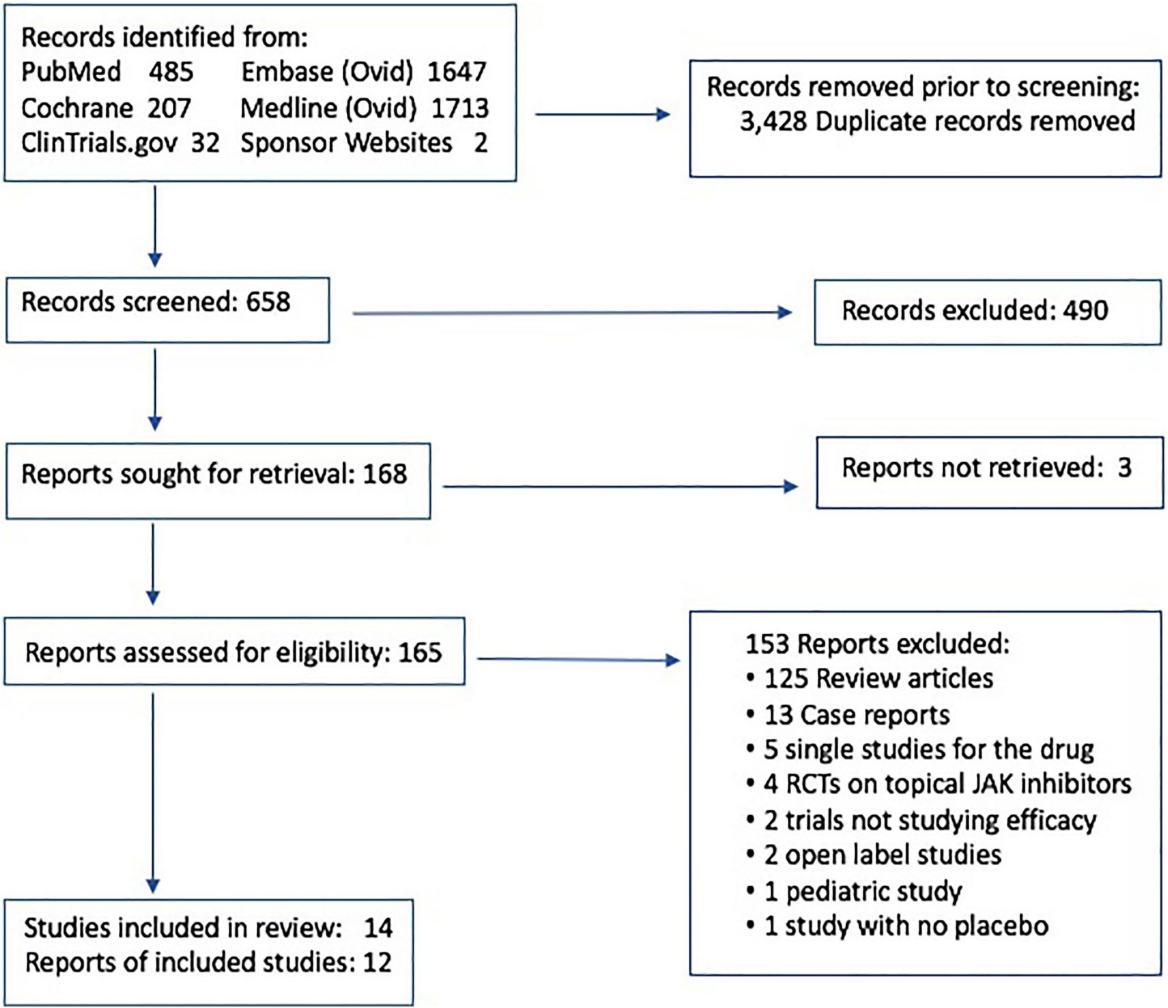
Preferred reporting items for systematic reviews and meta‐analyses flow diagram of the study

Only randomized controlled trials whose oral JAK inhibitors had results from more than one AD studies were included to allow for a comparison. Studies on topical JAK inhibitors were not included in this study. Studies included human subjects aged 12 and older and articles written in English. Abstracts, reviews, commentaries, case reports and case series were excluded. Two authors agreed on the articles. In case of discrepancy, the decision was deferred to the senior author. Figure 1 describes the methods of the systematic search.

### 3.3 Data extraction

Data extracted from the articles included the first author's name, publication year, clinical trial identifier, study design and study length. Patient characteristics included mean age, total number of participants, the key inclusion/exclusion criteria including percent body surface area (BSA), eczema area severity index (EASI) score and investigator global assessment (IGA) score. Study information included the treatment groups, the number of subjects in each group and the duration of treatment. Also, the primary and secondary endpoints including the number or percent of subjects achieving an IGA of clear or almost clear (0/1), EASI 50/75/90 (≥50%/75%/90% improvement of EASI score from baseline), pruritus numerical rating scale (NRS) improvement of ≥4 points, BSA and Scoring Atopic Dermatitis (SCORAD) compared to baseline, adverse events and serious adverse events were captured, as allowed by the availability of the data in the articles.

### 3.4 Bias evaluation

The risk of bias was assessed in accordance with the Cochrane Risk of Bias Tool.^12^ Each study was determined as having a low, high, or unclear risk of bias for random sequence generation (selection bias), allocation concealment (selection bias), blinding of participants and personnel (performance bias), blinding of outcome assessment (detection bias), selective reporting (reporting bias) and incomplete outcome data (attrition bias).

### 3.5 Statistical analysis

The meta‐analysis comparing the effect of JAK inhibitors against placebo was conducted using the metan procedure in STATA version 12.1. Separate meta‐analyses were run for each dose level of each compound for which multiple RCTs were available.

Relative risks (RRs) and standardized mean difference (SMD) with 95% confidence intervals (CIs) were used to evaluate the efficacy outcomes. RR was presented if the results were binary variables and SMD was used for data with continuous variables. Statistical significance was determined by a CI not containing 1 for the RR estimate, and a CI not containing 0 for the SMD estimate.

The heterogeneity was quantified using Cochran's *Q* and *I* ^2^ statistics measuring the percentage of variation between the studies.^13^ If the *I* ^2^ value was ≥50% and the *p* value was less than 0.05, heterogeneity was considered significant.

## 4 AD RESULTS

### 4.1 Search results

A PRISMA flowchart of the process of study selection is shown in Figure 1. Initially, a total of 4086 articles were retrieved. After screening the articles by title, abstract, and full‐text, 3921 articles were excluded including duplicates. Out of the 165 remaining relevant articles, were found to be original articles, of which 28 were excluded (13 case reports, 5 single studies for the specific JAK inhibitor, four RCTs on topical JAK inhibitors, two safety studies, two open‐label studies, one study evaluating paediatric subjects only, and one study without a placebo group). The final results included 12 eligible original articles with 14 RCTs for quantitative meta‐analysis.

### 4.2 Characteristics of eligible studies

The demographic data of the 14 clinical trials are seen in Table 1. The number of patients reaching the examined endpoints are shown in Table 2. Our meta‐analysis included 6653 patients with AD and assessed three JAK inhibitors. Of the 14 clinical trials, there were five, five and four RCTs for abrocitinib,^14^, ^15^, ^16^, ^17^, ^18^ baricitinib,^19^, ^20^, ^21^, ^22^ and upadacitinib,^23^, ^24^, ^25^ respectively. All drugs were administered orally in patients with moderate to severe AD. Moderate to severe AD was defined as IGA ≥3 and EASI ≥16 by all studies except one study defined moderate to severe AD as IGA ≥3 with an EASI ≥12. Three of the studies included concomitant topical corticosteroid treatment in addition to placebo or the JAK inhibitor.

**TABLE 1:** Demographic characteristics of the studies

Study	Treatment group	Age, mean (SD or IQR or range), year	Male sex *n* (%)	White, *n* (%)	Black, *n* (%)	Asian, *n* (%)	Other or not reported	Disease duration median (range or IQR), year	EASI, mean (SD or IQR)	IGA of 3 (Moderate)^a^	Pruritus NRS score, mean (SD or IQR)
Gooderham 2019 NCT02780167 18–75 years	Placebo	42.6 (15.1)	21 (37.5)	40 (71.4)	10 (17.9)	4 (7.1)	2 (3.6)	25.6 (1.1–67.1)	25.4 (12.9)	34 (61.8)	7.6 (1.8)
	Abrocitinib 100 mg	41.1 (15.6)	31 (55.4)	40 (71.4)	7 (12.5)	8 (14.3)	1 (1.8)	23.8 (1.1–66.7)	26.7 (11.8)	29 (52.7)	7.4 (2.2)
	Abrocitinib 200 mg	38.7 (17.6)	28 (50.9)	37 (67.3)	13 (23.6)	5 (9.1)	0	19.6 (1.9–68.8)	24.6 (13.5)	34 (63.0)	6.9 (2.7)
Silverberg 2020 NCT03575871 ≥12 years	Placebo	33.4 (13.8)	47 (60.3)	40 (51.3)	6 (7.7)	29 (37.2)	3 (3.9)	21.7 (14.3)	28.0 (10.2)	52 (66.7)	6.7 (1.9)
	Abrocitinib 100 mg	37.4 (15.8)	94 (59.5)	101 (63.9)	9 (5.7)	46 (29.1)	2 (1.2)	21.1 (14.8)	28.4 (11.2)	107 (67.7)	7.1 (1.6)
	Abrocitinib 200 mg	33.5 (14.7)	88 (56.8)	91 (58.7)	6 (3.9)	54 (34.8)	4 (2.6)	20.5 (14.8)	29.0 (12.4)	106 (68.4)	7.0 (1.6)
Simpson 2020 NCT03349060 ≥12 years	Placebo	31.5 (14.4)	49 (63.6)	62 (80.5)	6 (7.8)	6 (7.8)	3 (3.9)	22.5 (14.4)	28.7 (12.5)	46 (59.7)	7.0 (1.8)
	Abrocitinib 100 mg	32.6 (15.4)	90 (57.7)	113 (72.4)	15 (9.6)	26 (16.7)	2 (1.3)	24.9 (16.1)	31.3 (13.6)	92 (59.0)	6.9 (2.0)
	Abrocitinib 200 mg	33.0 (17.4)	81 (52.6)	104 (67.5)	11 (7.1)	26 (16.7)	13 (8.4)	22.7 (14.5)	30.6 (14.1)	91 (59.1)	7.1 (1.9)
Bieber 2021 NCT03720470 ≥18 years	Placebo	37.4 (15.2)	77 (58.8)	87 (66.4)	6 (4.6)	31 (23.7)	7 (5.3)	21 (14.4)	31.0 (12.6)	88 (67.2)	7.1 (1.8)
	Abrocitinib 100 mg	37.3 (14.8)	120 (50.4)	182 (76.5)	6 (2.5)	48 (20.2)	2 (0.8)	22.7 (16.3)	30.3 (13.5)	153 (64.3)	7.1 (1.7)
	Abrocitinib 200 mg	38.8 (14.5)	104 (46.0)	161 (71.2)	9 (4.0)	53 (23.5)	3 (1.3)	23.4 (15.6)	32.1 (13.1)	138 (61.1)	7.6 (1.5)
Eichenfield 2021 NCT03796676 12–17 years.o.	Placebo	14.0 (13.5–16.5)	44 (45.8)	56 (58.3)	3 (3.1)	32 (33.3)	5 (5.1)	10.5 (4.8)	29.2 (12.7)	57 (59.4)	7.2 (1.7)
	Abrocitinib 100 mg	16.0 (14.0–17.0)	45 (47.4)	52 (54.7)	9 (9.5)	31 (32.6)	3 (3.2)	9.8 (5.4)	31.0 (12.8)	57 (60.0)	7.0 (1.8)
	Abrocitinib 200 mg	15.0 (13.0–16.0)	56 (59.6)	52 (55.3)	5 (5.3)	31 (33.0)	6 (6.5)	9.7 (5.3)	29.5 (12.2)	61 (64.9)	6.8 (2.0)
Guttman‐Yassky 2018 NCT02576938 ≥18 years	Placebo	35 (28.0–48.0)	24 (49.0)	23 (46.9)	7 (14.3)	16 (32.7)	3 (6.1)	17.7 (7.3–29.5)	22.1 (15.3–28.0)	ND	7 (6, 8)
	Baricitinib 2 mg	42 (26.0–52.0)	22 (59.5)	20 (54.1)	9 (24.3)	8 (21.6)	0	26.4 (18.3–40.5)	22.1 (16.8–32.3)	ND	6 (5, 8)
	Baricitinib 4 mg	32.5 (26.0–48.0)	22 (57.9)	18 (47.4)	9 (23.7)	9 (23.7)	2 (5.3)	22.0 (6.4–30.7)	19.5 (13.7–25.9)	ND	6.5 (4, 8)
Simpson 2020 (BREEZE‐AD 1) NCT03334396 ≥18 years	Placebo	35 (12.6)	148 (59.4)	147 (59.5)	ND	73 (29.6)	ND	26 (15.5)	32 (13.0)	144 (57.8)	6.7 (2.0)
	Baricitinib 1 mg	36 (12.4)	78 (61.4)	74 (58.3)	ND	40 (31.5)	ND	27 (14.9)	29 (11.8)	74 (58.3)	6.1 (2.1)
	Baricitinib 2 mg	35 (13.7)	82 (66.7)	75 (61.0)	ND	35 (28.5)	ND	25 (14.6)	31 (11.7)	71 (57.7)	6.4 (2.2)
	Baricitinib 4 mg	37 (12.9)	83 (66.4)	70 (56.5)	ND	41 (33.1)	ND	25 (14.9)	32 (12.7)	74 (59.2)	6.5 (2.0)
Simpson 2020 (BREEZE‐AD 2) NCT03334422 ≥18 years	Placebo	35 (13.0)	154 (63.1)	169 (69.3)	ND	71 (29.5)	ND	25 (13.9)	33 (12.8)	123 (50.4)	6.8 (2.2)
	Baricitinib 1 mg	33 (10.0)	80 (4.0)	85 (68.0)	ND	36 (28.8)	ND	24 (12.7)	33 (12.7)	62 (49.6)	6.4 (2.2)
	Baricitinib 2 mg	36 (13.2)	65 (52.8)	85 (69.1)	ND	37 (30.1)	ND	24 (13.8)	35 (16.0)	61 (49.6)	6.6 (2.2)
	Baricitinib 4 mg	34 (14.1)	82 (66.7)	82 (66.7)	ND	38 (30.9)	ND	23 (14.8)	33 (12.7)	60 (48.8)	6.6 (2.2)
Reich 2020 NCT03733301 ≥18 years	Placebo	33.7 (13.2)	71 (65.0)	46 (42)	ND	57 (52)	6 (6)	22.0 (12.2)	28.5 (12.3)	60 (55.6)	7.4 (1.7)
	Baricitinib 2 mg	33.8 (12.8)	70 (64.2)	50 (46)	ND	57 (52)	2 (2)	24.6 (14.8)	29.3 (11.9)	59 (54.1)	7.0 (2.1)
	Baricitinib 4 mg	33.9 (11.4)	75 (67.6)	54 (49)	ND	54 (49)	3 (3)	25.5 (13.2)	30.9 (12.6)	61 (55.0)	7.0 (2.0)
Simpson 2021 NCT03435081 ≥18 years	Placebo	39 (17)	80 (54.4)	80 (54.4)	24 (16.3)	33 (22.4)	9 (6.1)	23 (17)	27.0 (11)	86 (58.5)	7.0 (2.4)
	Baricitinib 1 mg	40 (17)	75 (51.0)	86 (58.5)	26 (17.7)	26 (17.7)	8 (5.4)	24 (17)	27.2 (12)	85 (57.8)	7.2 (2.0)
	Baricitinib 2 mg	40 (15)	69 (47.3)	85 (58.2)	30 (20.5)	22 (15.1)	9 (6.2)	24 (16)	26.6 (11)	85 (58.2)	7.3 (2.1)
Guttman‐Yassky 2020 NCT02925117 18–75 years.o.	Placebo	39.9 (1.5)	24 (58.5)	28 (68.3)	6 (14.6)	7 (17.1)	0	26.8 (18.8)	32.6 (14.5)	18 (43.9)	6.5 (1.9)
	Upadacitinib 15 mg	38.5 (15.2)	30 (71.4)	21 (50.0)	10 (23.8)	9 (21.4)	2 (4.8)	22.6 (15.8)	31.4 (12.3)	19 (45.2)	6.4 (1.7)
	Upadacitinib 30 mg	39.9 (15.3)	22 (52.3)	23 (54.8)	6 (14.3)	13 (31.0)	0	24.2 (13.6)	28.2 (11.6)	31 (73.8)	6.3 (2.1)
Guttman‐Yassky 2021 (measure up 1) NCT03569293 12–75 years.o.	Placebo	34.4 (12–75)	144 (51.2)	182 (64.8)	21 (7.5)	69 (24.6)	9 (3.2)	21.3 (15.3)	28.8 (12.6)	156 (55.5)	7.5 (1.8)
	Upadacitinib 15 mg	34.1 (12–74)	157 (55.9)	182 (64.8)	26 (9.3)	63 (22.4)	10 (3.6)	20.5 (15.9)	30.6 (12.8)	154 (54.8)	7.4 (1.8)
	Upadacitinib 30 mg	33.6 (12–75)	155 (54.4)	191 (67.0)	8 (2.8)	71 (24.9)	15 (5.3)	20.4 (14.3)	29.0 (11.1)	154 (54.0)	7.5 (1.7)
Guttman‐Yassky 2021 (measure up 2) NCT03607422 12–75 years.o.	Placebo	33.4 (13–71)	154 (55.4)	195 (70.1)	16 (5.8)	56 (20.1)	11 (4.0)	21.1 (13.6)	29.1 (12.1)	125 (45.0)	7.5 (1.9)
	Upadacitinib 15 mg	33.3 (12–74)	155 (56.2)	184 (66.7)	17 (6.2)	65 (23.6)	26 (3.6)	18.8 (13.3)	28.6 (11.7)	126 (45.7)	7.2 (1.8)
	Upadacitinib 30 mg	34.1 (12–75)	162 (57.4)	198 (70.2)	18 (6.4)	62 (22.0)	4 (1.4)	20.8 (14.3)	29.7 (12.2)	126 (44.7)	7.4 (1.7)
Reich 2021 NCT03568318 12–75 years.o.	Placebo	34.3 (12–75)	178 (58.6)	225 (74.0)	18 (5.9)	60 (19.7)	1 (0.3)	24.3 (15.2)	30.3 (13.0)	141 (46.4)	7.1 (1.6)
	Upadacitinib 15 mg	32.5 (13–74)	179 (59.7)	204 (68.0)	19 (6.3)	64 (21.3)	13 (4.3)	22.9 (13.9)	29.2 (11.8)	143 (47.7)	7.1 (1.8)
	Upadacitinib 30 mg	35.5 (12–72)	190 (64.0)	218 (73.4)	13 (4.4)	61 (20.5)	5 (1.7)	23.1 (16.1)	29.7 (11.8)	140 (47.1)	7.4 (1.6)

Abbreviations: *%, percent of patients; EASI, eczema area and skin severity index; IGA, investigator global assessment; IQR, interquartile range; n, number of patients; NRS, numerical rating scale; SD, standard deviation.*

^a^ All subjects had either IGA = 3 or 4.

**TABLE 2:** Efficacy results from the examined endpoints included in the meta‐analysis

Author		Placebo	200 mg abrocitinib	100 mg abrocitinib
Gooderham 2019	EASI‐75 at week 12, *n* (%)	8 (15.4)	31 (64.6)	22 (40.7)
	% Change in EASI score at week 12, LSM (SE)	−35.2 (6.6)	−82.6 (6.2)	−59 (6.2)
	IGA 0/1 at week 12, *n* (%)	3 (5.8)	21 (43.8)	16 (29.6)
	Pruritus NRS ≥4 point improvement at week 12, *n* (%)	13 (25.5)	28 (63.6)	25 (50.0)
Silverberg 2020	EASI‐75 at week 12, *n* (%)	8 (10.4)	94 (61.0)	69 (44.5)
	%Change in EASI score, LSM (SE)	ND	ND	ND
	IGA 0/1 at week 12, *n* (%)	7 (9.1)	59 (38.1)	44 (28.4)
	Pruritus NRS ≥4 point improvement at week 12, *n* (%)	9 (11.5)	85 (55.3)	71 (45.2)
Simpson 2020	EASI‐75 at week 12, *n* (%)	9 (11.7)	96 (62.3)	62 (39.7)
	% Change in EASI score, LSM (95% CI)	−8.2 (−10.9/−5.5)	−22.3 (−24.1/−20.4)	−16.6 (−18.4/−14.7)
	IGA 0/1 at week 12, *n* (%)	6 (7.8)	67 (43.5)	37 (23.7)
	Pruritus NRS ≥4 point improvement at week 12, *n* (%)	11 (14.3)	84 (54.5)	55 (35.3)
Bieber 2021	EASI‐75 at week 12, *n* (%)	35 (27.1)	154 (70.3)	138 (58.7)
	% Change in EASI score	ND	ND	ND
	IGA 0/1 at week 12, *n* (%)	18 (14.0)	106 (48.4)	86 (36.6)
	Pruritus NRS ≥4 point improvement at week 12, *n* (%)	35 (28.9)	137 (63.1)	105 (47.5)
Eichenfield 2021	EASI‐75 at week 12, *n* (%)	39 (41.5)	67 (72.0)	61 (68.5)
	% Change in EASI score, LSM (95% CI)	−63.7 (−69.5/‐57.9)	−80.6 (−86.5/−74.8)	−77.3 (−83.1/−71.5)
	IGA 0/1 at week 12, *n* (%)	23 (24.5)	43 (46.2)	37 (41.6)
	Pruritus NRS ≥4 point improvement at week 12, *n* (%)	25 (29.8)	41 (55.4)	40 (52.6)

		**Placebo**	**1 mg baricitinib**	**2 mg baricitinib**	**4 mg baricitinib**
Guttman‐Yassky 2018	EASI‐75 at week 16, *n* (%)	ND	ND	ND	ND
	% Change in EASI score at week 16, LSM (SE)	−45.9 (5.9)	ND	−64.2 (6.2)	−64.7 (6.2)
	IGA 0/1 at week 16, *n* (%)	8 (16.3)	ND	22 (59.5)	21 (55.3)
	Pruritus NRS ≥4 point improvement at week 12, *n* (%)	ND	ND	ND	ND
Simpson 2020 (BREEZE‐AD1)	EASI‐75 at week 16, *n* (%)	22 (8.8)	22 (17.3)	23 (18.7)	31 (24.8)
	% Change in EASI score at week 16, LSM (SE)	−34.8 (3.6)	−48.2 (4.5)	−51.9 (4.3)	−59.36 (3.8)
	IGA 0/1 at week 16, *n* (%)	12 (4.8)	15 (11.8)	14 (11.4)	21 (16.8)
	Pruritus NRS ≥4 point improvement at week 12, *n* (%)	16 (7.2)	11 (10.5)	12 (12.0)	23 (21.5)
Simpson 2020 (BREEZE‐AD2)	EASI‐75 at week 16, *n* (%)	15 (6.1)	16 (12.8)	22 (17.9)	26 (21.1)
	% Change in EASI score at week 16, LSM (SE)	−28.9 (4.3)	−41.7 (5.3)	−54.8 (5.0)	−54.8 (4.6)
	IGA 0/1 at week 16, *n* (%)	11 (4.5)	11 (8.8)	13 (10.6)	17 (13.8)
	Pruritus NRS ≥4 point improvement at week 12, *n* (%)	10 (4.7)	6 (6.0)	16 (15.1)	20 (18.7)
Reich 2020	EASI‐75 at week 16, *n* (%)	25 (22.9)	ND	47 (43.1)	53 (47.7)
	% Change in EASI score at week 16, LSM(SE)	−45.1 (3.8)	ND	−58.2 (3.7)	−67.2 (3.7)
	IGA 0/1 at week 16, *n* (%)	16 (14.7)	ND	26 (23.9)	34 (30.6)
	Pruritus NRS ≥4 point improvement at week 12, *n* (%)	21 (20.2)	ND	37 (38.1)	44 (44.0)
Simpson 2021	EASI‐75 at week 16, *n* (%)	12 (8.2)	19 (12.9)	43 (29.5)	ND
	% Change in EASI score at week 16, LSM (SE)	−34.1 (5.5)	−46.7 (4.5)	−54.4 (4.2)	ND
	IGA 0/1 at week 16, *n* (%)	8 (5.4)	19 (12.9)	35 (24.0)	ND
	Pruritus NRS ≥4 point improvement at week 12, *n* (%)	7 (5.7)	21 (15.9)	33 (25.2)	ND

		**Placebo**	**15 mg upadacitinib**	**30 mg upadacitinib**
Guttman‐Yassky 2020	EASI‐75 at week 16, *n* (%)	4 (9.8)	22 (52.4)	29 (69.0)
	% Change in EASI score at week 16, LSM (SE)	23.0 (6.4)	61.7 (6.1)	74.4 (6.1)
	IGA 0/1 at week 16, *n* (%)	1 (2.4)	13 (31.0)	21 (50.0)
	Pruritus NRS ≥4 point improvement at week 12, *n* (%)	2 (5.7)	19 (59.4)	19 (52.8)
Guttman‐Yassky 2021 (measure up 1)	EASI‐75 at week 16, *n* (%)	46 (16.3)	196 (69.6)	227 (79.7)
	% Change in EASI score at week 16, LSM (95% CI)	−40.7 (−45.2/−36.2)	−80.2 (−84.0/−76.5)	−87.7 (−91.4/−84.1)
	IGA 0/1 at week 16, *n* (%)	24 (8.4)	135 (48.1)	177 (62.0)
	Pruritus NRS ≥4 point improvement at week 12, *n* (%)	32 (11.8)	143 (52.2)	168 (60.0)
Guttman‐Yassky 2021 (measure up 2)	EASI‐75 at week 16, *n* (%)	37 (13.3)	166 (60.1)	206 (72.9)
	% Change in EASI score at week 16, LSM (95% CI)	−34.5 (−39.6/‐29.4)	−74.1 (−78.4/‐69.8)	−84.7 (−88.9/‐80.4)
	IGA 0/1 at week 16, *n* (%)	13 (4.7)	107 (38.8)	147 (52.0)
	Pruritus NRS ≥4 point improvement at week 12, *n* (%)	25 (9.1)	113 (41.9)	167 (59.6)
Reich 2021	EASI‐75 at week 16, *n* (%)	80 (26.4)	194 (64.6)	229 (77.1)
	% Change in EASI score at week 16, LSM (95% CI)	−45.9 (−41.6/−50.1)	−78.0 (−74.1/−81.9)	−87.3 (−83.4/−91.2)
	IGA 0/1 at week 16, *n* (%)	33 (10.9)	119 (39.6)	174 (58.6)
	Pruritus NRS ≥4 point improvement at week 12, *n* (%)	44 (15.0)	149 (51.7)	186 (63.9)

Abbreviations: %, percent of patients; CI, confidence interval; EASI, eczema area and skin severity index; IGA, investigator global assessment; LSM, least squares mean; n, number of patients; ND, no data; NRS, numerical rating scale; SE, standard error.

### 4.3 Risk of bias assessment

**FIGURE 2:**
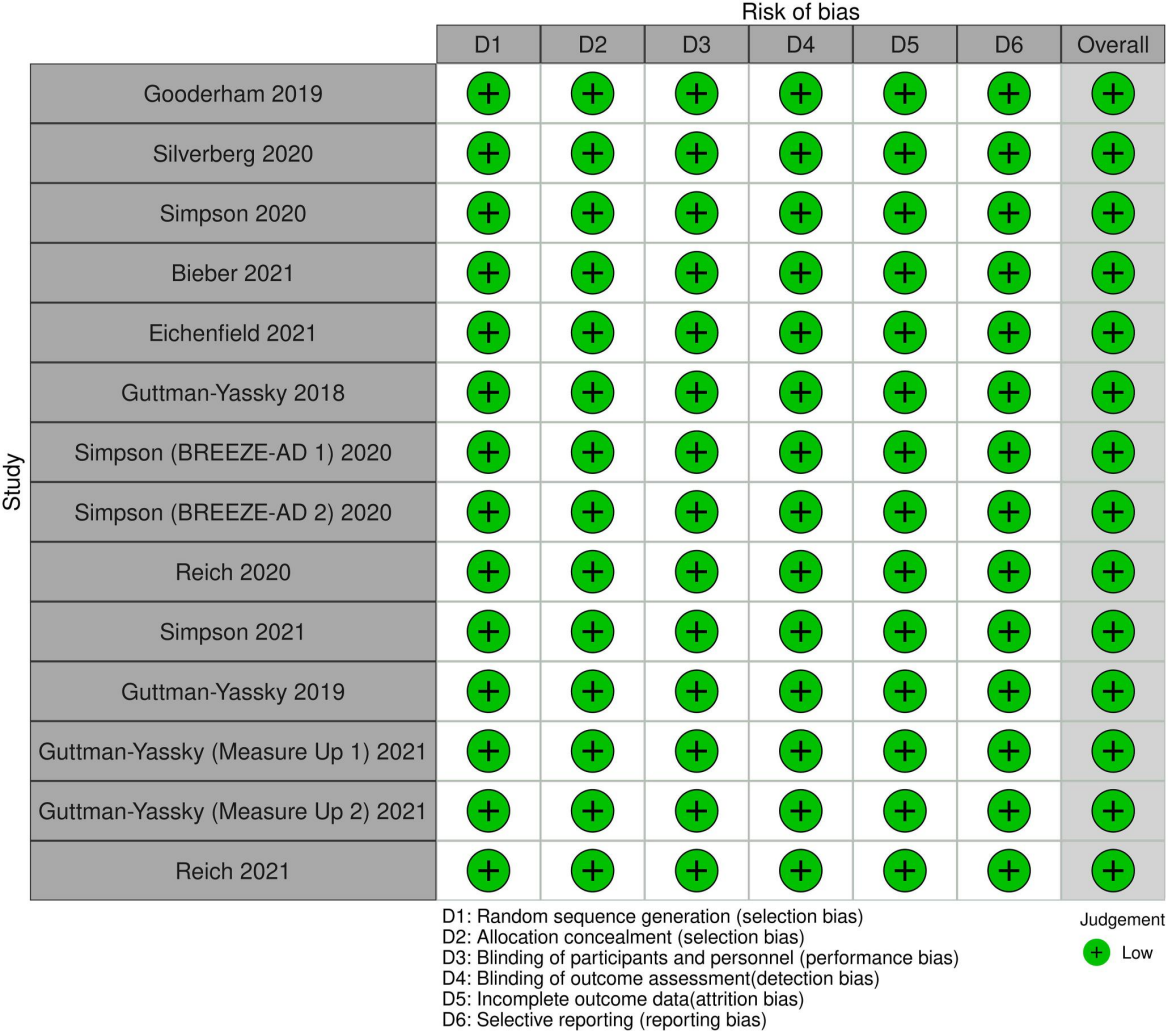
Risk of bias assessment

The Risk‐of‐Bias Visualisation tool was used to create the plot of bias (Figure 2).^26^ All 14 RCTs were found to have a low risk of bias.

### 4.4 Efficacy outcomes

#### 4.4.1 EASI‐75 Response

Thirteen RCTs (6927 patients) reporting EASI‐75 responses were analysed. Seven separate meta‐analyses were performed for patients on abrocitinib 100 mg, abrocitinib 200 mg, baricitinib 1 mg, baricitinib 2 mg, baricitinib 4 mg, upadacitinib 15 mg, and upadacitinib 30 mg (Figure 3a–g). Patients taking abrocitinib 100 mg (RR = 2.33; 95% CI = 1.92–2.82), abrocitinib 200 mg (RR = 3.00; 95% CI = 2.49–3.62), baricitinib 1 mg (RR = 1.87; 95% CI = 1.30–2.69), baricitinib 2 mg (RR = 2.46; 95% CI = 1.89–3.18), baricitinib 4 mg (RR = 2.57, 95% CI = 1.95–3.38), upadacitinib 15 mg (RR = 3.48, 95% CI = 3.01–4.03), and upadacitinib 30 mg (RR = 4.14, 95% CI = 3.59–4.77) had a significantly higher rate of achieving EASI‐75 than patients in the placebo group (Table 3). Significant heterogeneity was observed for abrocitinib 100 mg (*I* ^2^ = 66.1%, *p* = 0.019), abrocitinib 200 mg (*I* ^2^ = 83.4%, *p* = 0.000), upadacitinib 15 mg (*I* ^2^ = 81.8%, *p* = 0.001) and upadacitinib 30 mg (*I* ^2^ = 82.9%, *p* = 0.001). No significant heterogeneity was observed for baricitinib 1 mg (*I* ^2^ = 0.0%, *p* = 0.838), 2 mg (*I* ^2^ = 25.7%, *p* = 0.257), or 4 mg (*I* ^2^ = 5.7%, *p* = 0.346).

FIGURE 3Forest plots for the proportion of patients on oral abrocitinib 100 mg (a), oral abrocitinib 200 mg (b), oral baricitinib 1 mg (c), oral baricitinib 2 mg (d), oral baricitinib 4 mg (e), oral upadacitinib 15 mg (f), and oral upadacitinib 30 mg (g) achieving EASI‐75 response compared to patients on placebo

**Figure:**
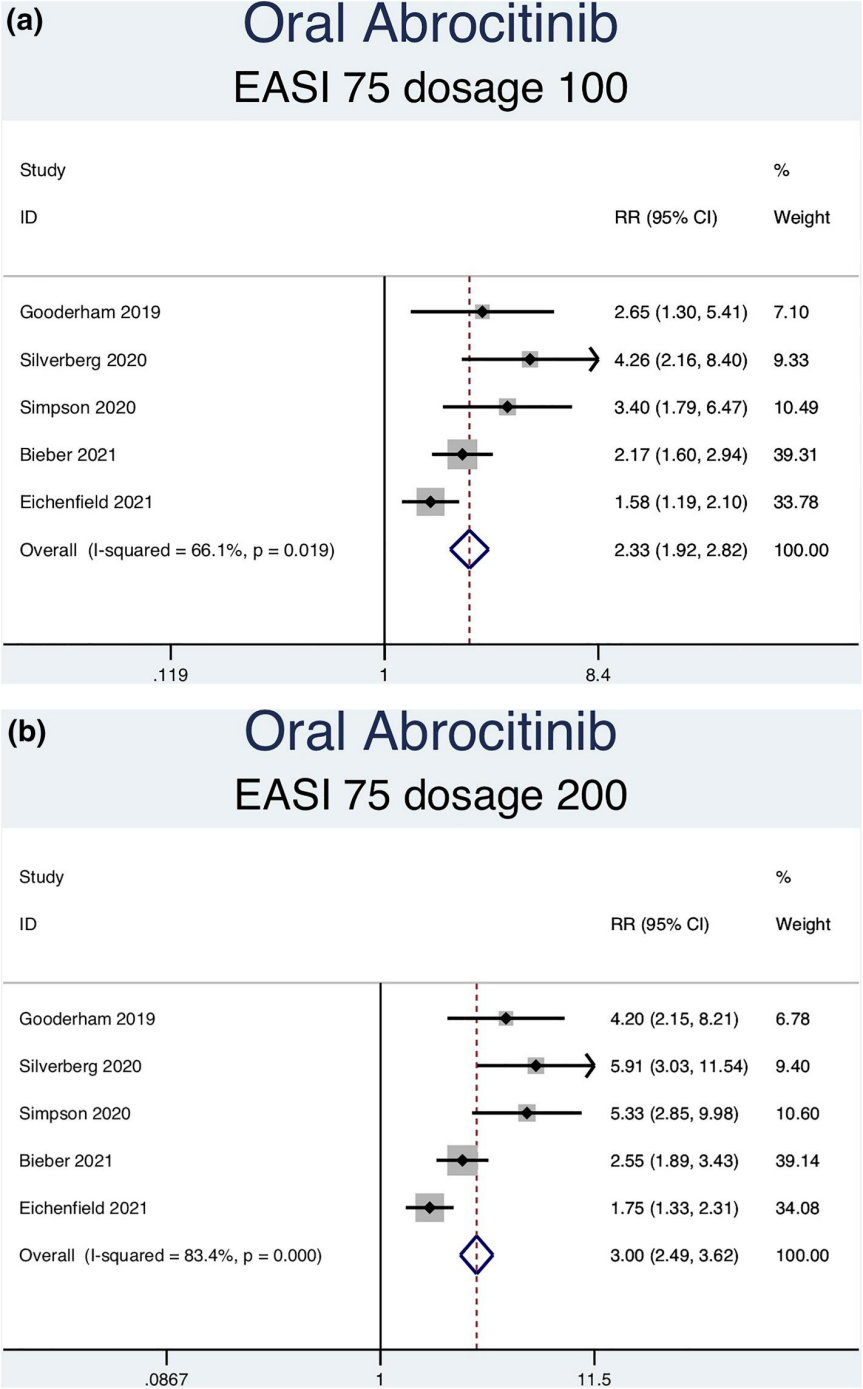

**Figure:**
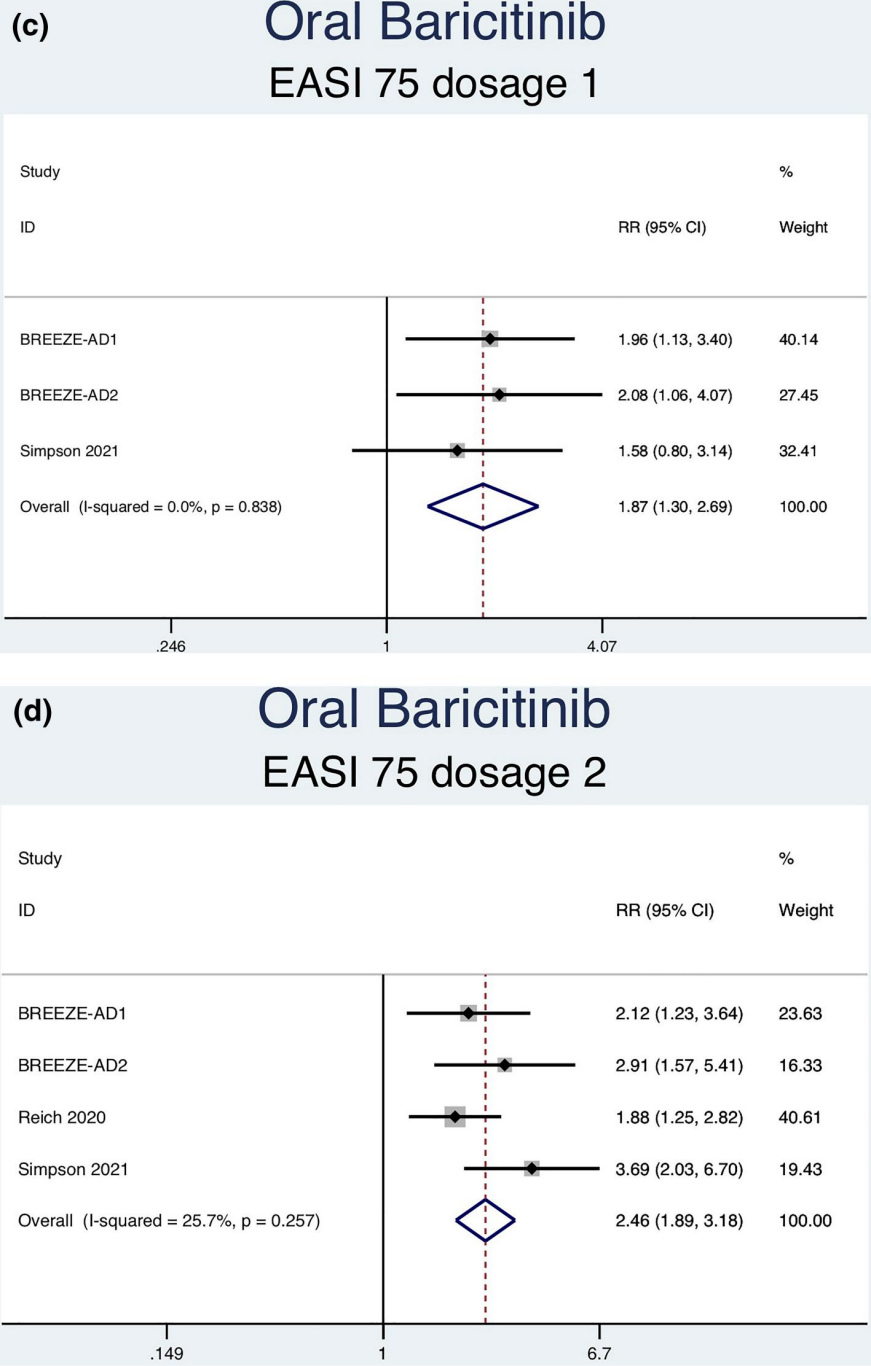

**Figure:**
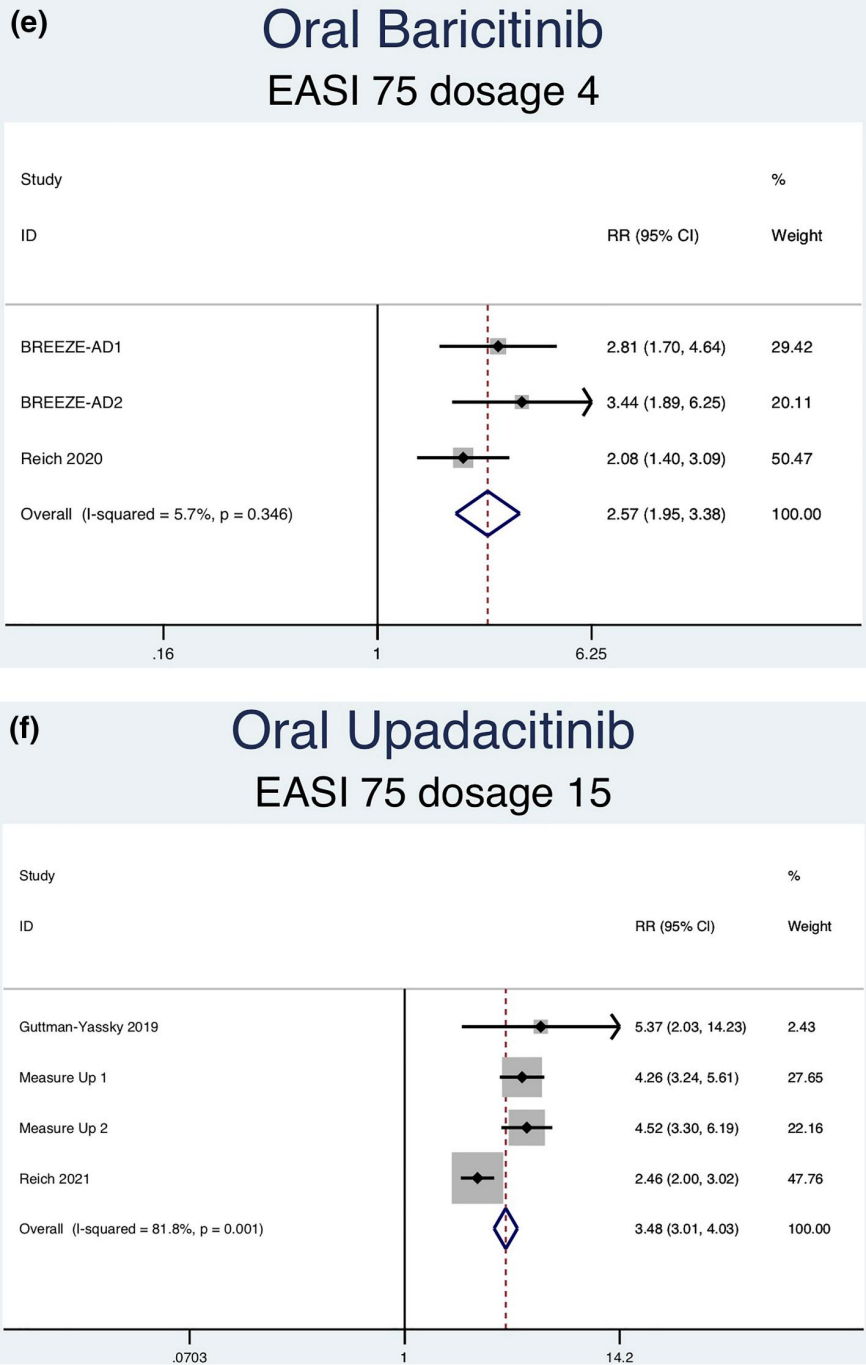

**Figure:**
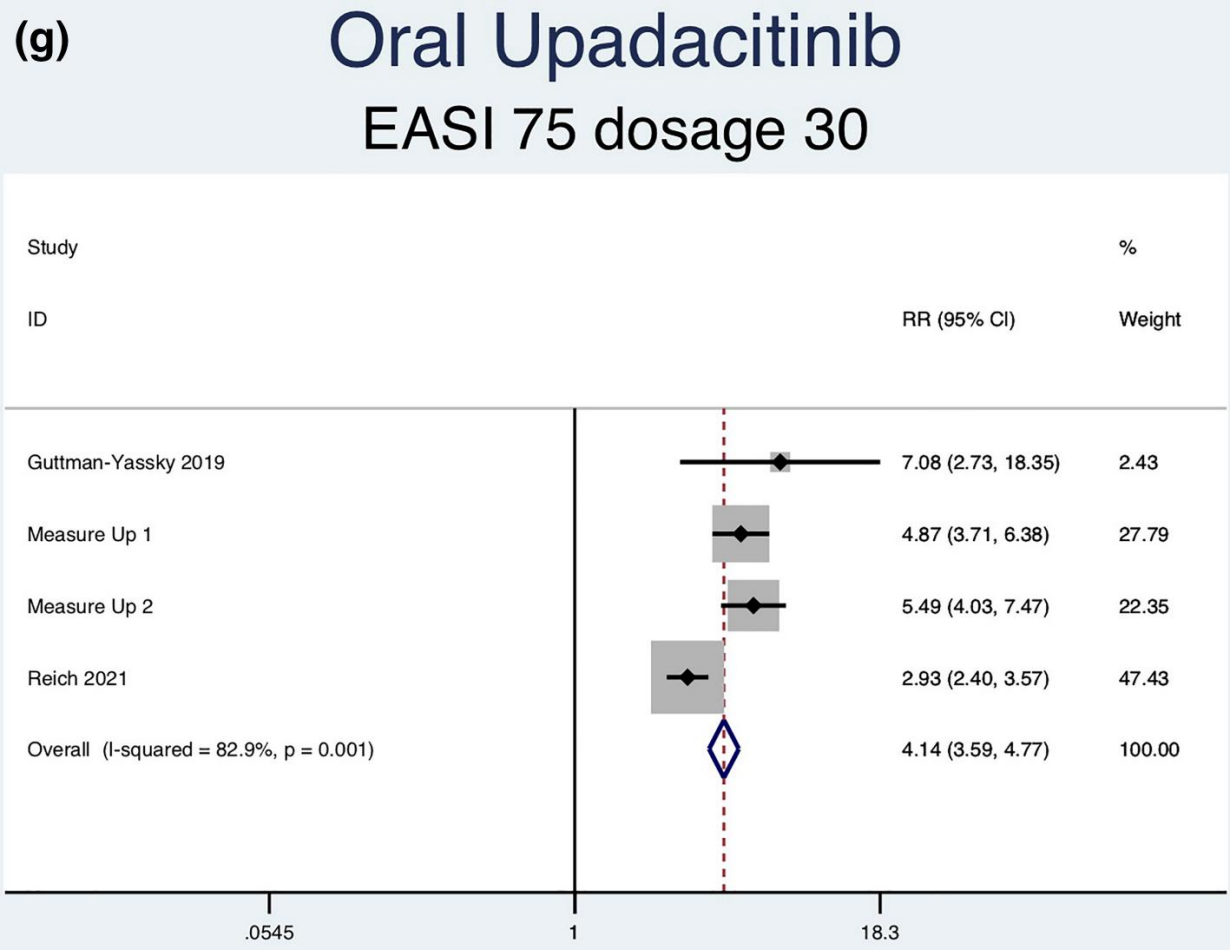

**TABLE 3:** Relative risk (RR) values for each of drug dosages

	EASI 75 RR (95% CI)	% Change in EASI SMD (95% CI)	IGA 0/1 RR (95% CI)	Pruritus NRS score ≥4 point improvement RR (95% CI)
Abrocitinib 100 mg	2.33 (1.92–2.82)	−0.58 (−0.76 to −0.40)	2.52 (1.92–3.30)	2.02 (1.65–2.48)
Abrocitinib 200 mg	3.00 (2.49–3.62)	−0.92 (−1.11 to −0.74)	3.46 (2.66–4.50)	2.63 (2.15–3.20)
Baricitinib 1 mg	1.87 (1.30–2.69)	−0.22 (−0.34 to −0.09)	2.27 (1.45–3.55)	1.74 (1.09–2.79)
Baricitinib 2 mg	2.46 (1.89–3.18)	−0.36 (−0.47 to −0.25)	2.65 (1.95–3.59)	2.35 (1.72–3.22)
Baricitinib 4 mg	2.57 (1.95–3.38)	−0.47 (−0.60 to −0.35)	2.80 (2.03–3.86)	2.61 (1.90–3.60)
Upadacitinib 15 mg	3.48 (3.01–4.03)	−0.99 (−1.09 to −0.89)	5.30 (4.19–6.71)	4.14 (3.41–5.03)
Upadacitinib 30 mg	4.14 (3.59–4.77)	−1.25 (−1.35 to −1.15)	7.31 (5.81–9.21)	5.24 (4.34–6.34)

Abbreviations: CI, confidence interval; EASI, eczema area and skin severity index; IGA, investigator global assessment; SMD, standardized mean difference.

Twelve RCTs (5822 patients) reporting percent change in EASI scores were analysed. Seven separate meta‐analyses were performed (Figure 4a–g). All groups analysed [abrocitinib 100 mg (SMD = −0.58, 95% CI = −0.76 to −0.40), abrocitinib 200 mg (SMD = −0.92, 95% CI = −1.11 to −0.74), baricitinib 1 mg (SMD = −0.22, 95% CI = −0.34 to −0.09), baricitinib 2 mg (SMD = −0.36, 95% CI = −0.47 to −0.25), baricitinib 4 mg (SMD = −0.47, 95% CI = −0.60 to −0.35), upadacitinib 15 mg (SMD = −0.99, 95% CI = −1.09 to −0.89) and upadacitinib 30 mg (SMD = −1.25, 95% CI = −1.35 to −1.15)] showed a statistically significant decrease in percent EASI score compared to placebo. Significant heterogeneity was seen in abrocitinib 200 mg (*I* ^2^ = 78.6%, *p* = 0.009). No significant heterogeneity was seen in any of the other groups [abrocitinib 100 mg (*I* ^2^ = 0.0%, *p* = 0.444), baricitinib 1 mg (*I* ^2^ = 0.0%, *p* = 0.955), baricitinib 2 mg (*I* ^2^ = 0.0%, *p* = 0.950), baricitinib 4 mg (*I* ^2^ = 0.0%, *p* = 0.871), upadacitinib 15 mg (*I* ^2^ = 1.2%, *p* = 0.386) and upadacitinib 30 mg (*I* ^2^ = 0.0%, *p* = 0.580)].

FIGURE 4Forest plots for the percent change of EASI score in patients on oral abrocitinib 100 mg (a), oral abrocitinib 200 mg (b), oral baricitinib 1 mg (c), oral baricitinib 2 mg (d), oral baricitinib 4 mg (e), oral upadacitinib 15 mg (f), and oral upadacitinib 30 mg (g) compared to patients on placebo

**Figure:**
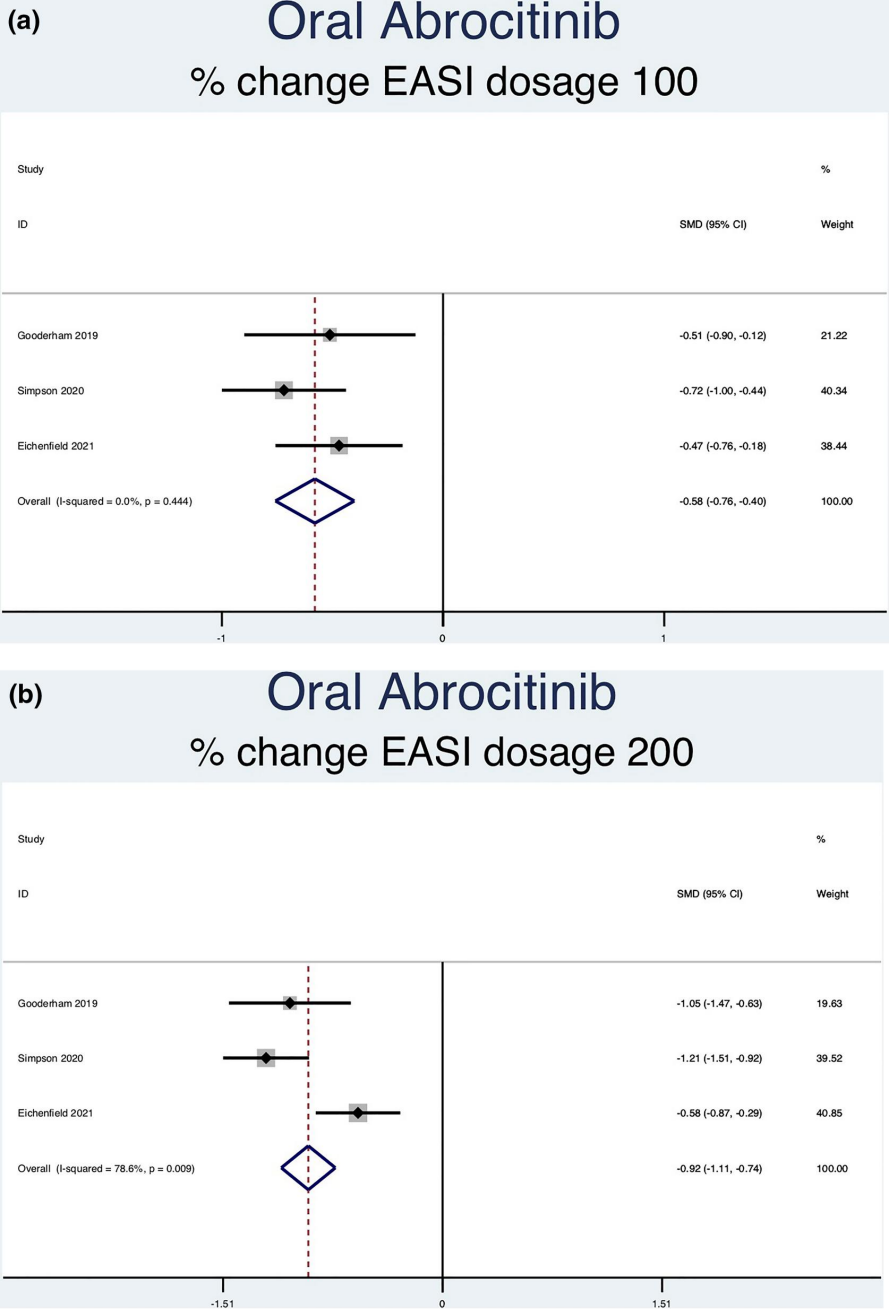

**Figure:**
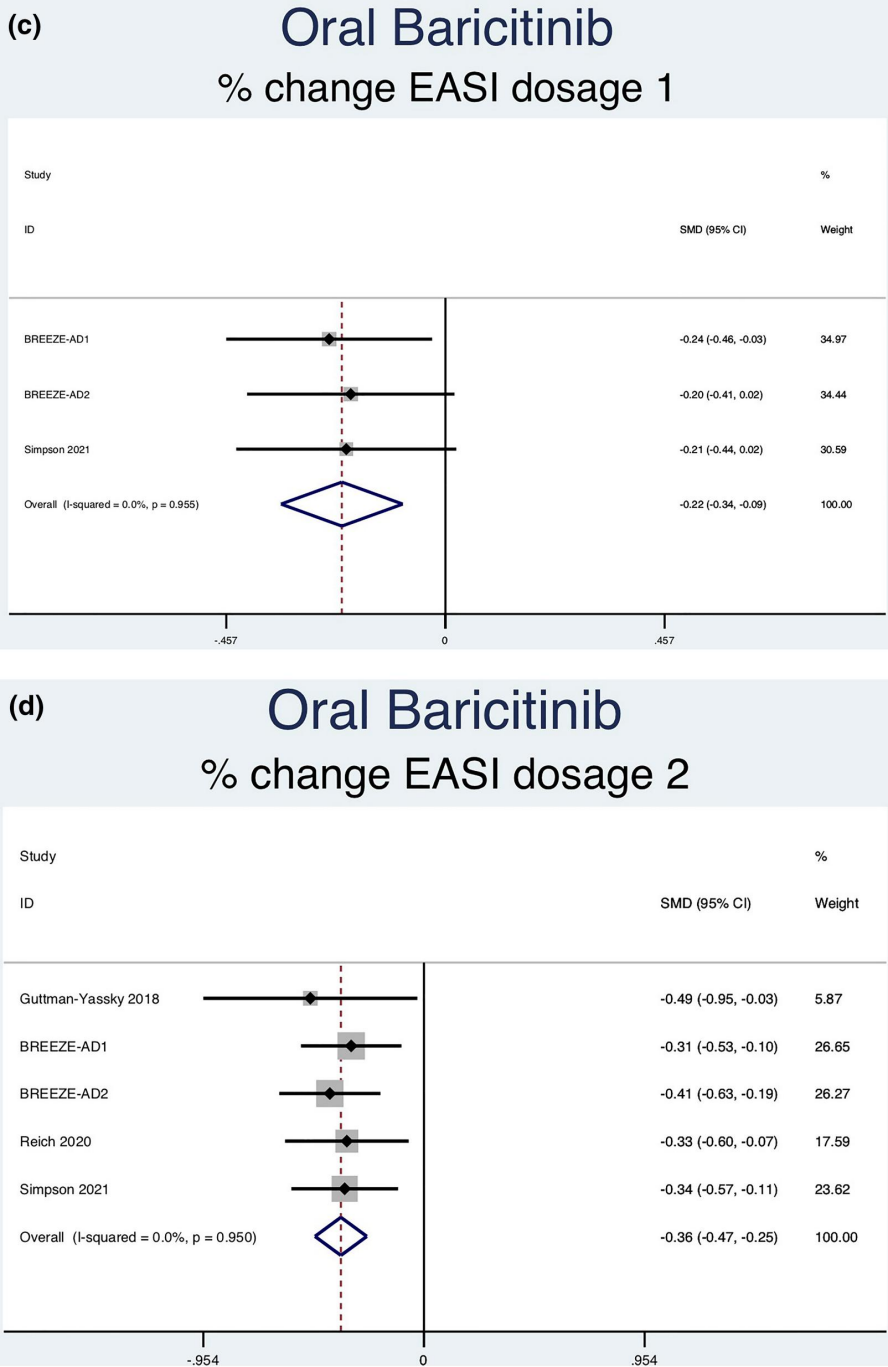

**Figure:**
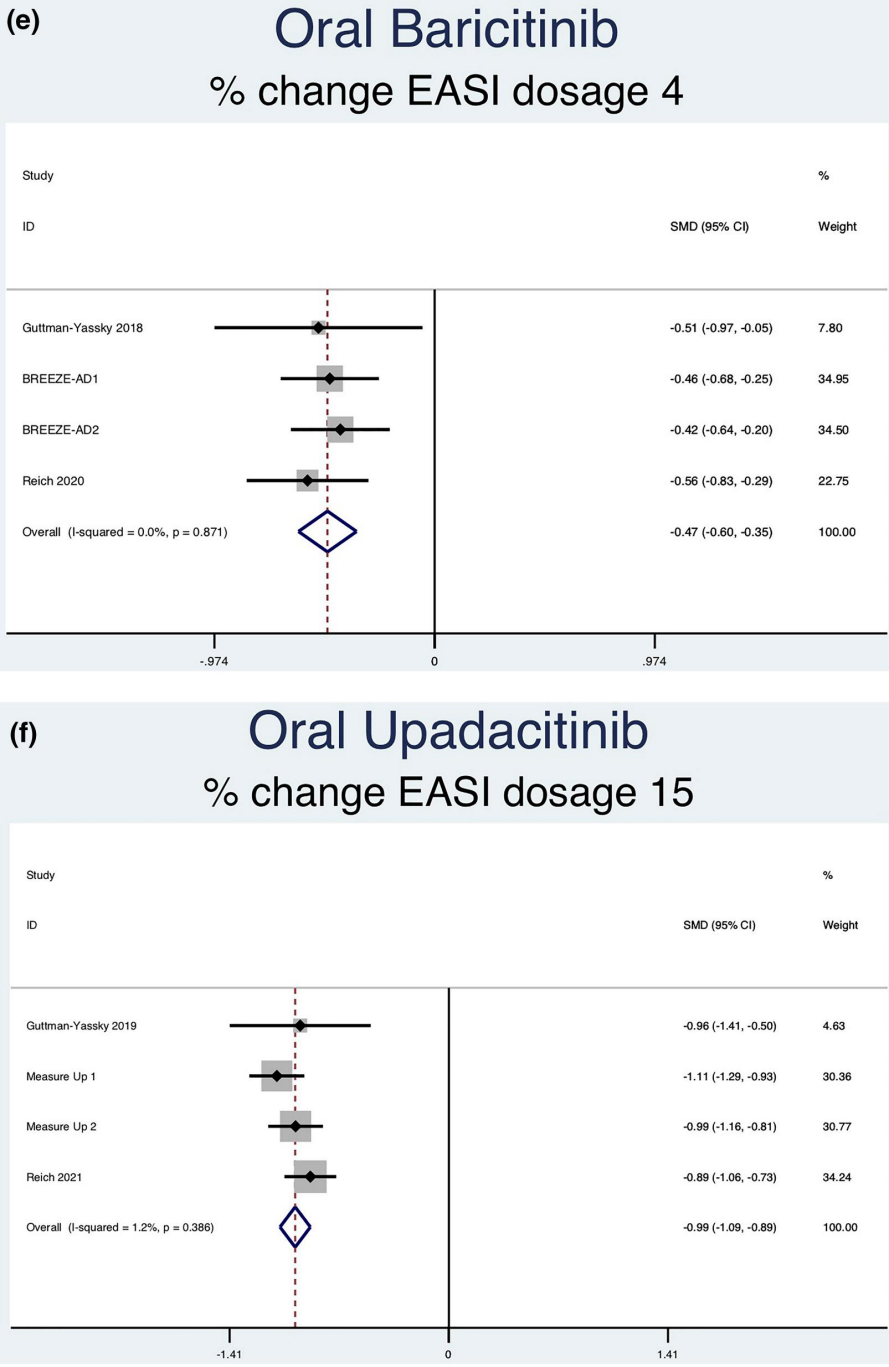

**Figure:**
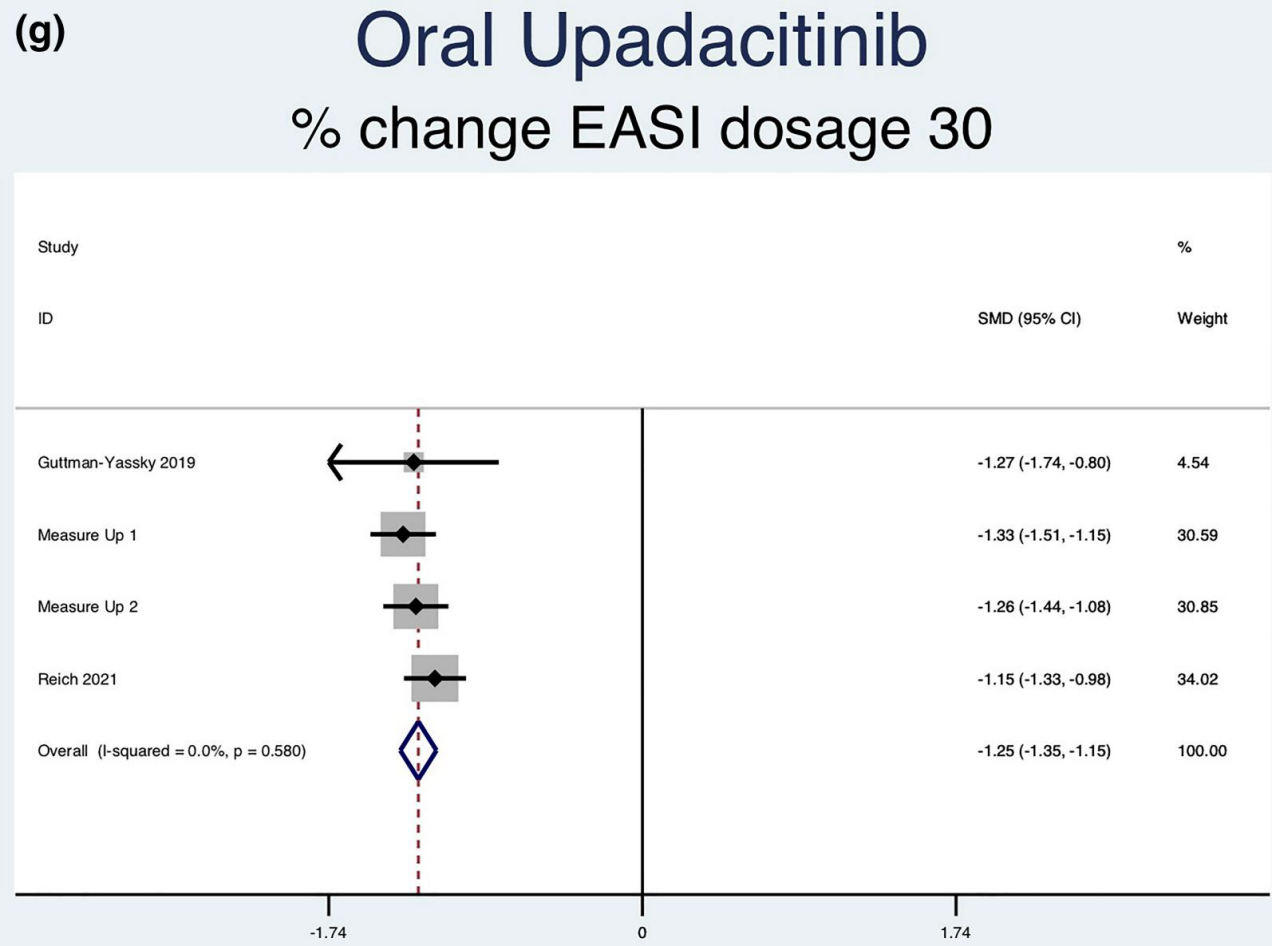

Fourteen RCTs (7051 patients) reporting IGA responses of 0 or 1 as an outcome were analysed. Seven separate meta‐analyses (Figure 5a–g) were performed for patients on abrocitinib 100 mg, abrocitinib 200 mg, baricitinib 1 mg, baricitinib 2 mg, baricitinib 4 mg, upadacitinib 15 mg and upadacitinib 30 mg. All groups [abrocitinib 100 mg (RR = 2.52, 95% CI = 1.92–3.30), abrocitinib 200 mg (RR = 3.46, 95% CI = 2.66–4.50), baricitinib 1 mg (RR = 2.27, 95% CI = 1.45–3.55), baricitinib 2 mg (RR = 2.65, 95% CI = 1.95–3.59), baricitinib 4 mg (RR = 2.80, 95% CI = 2.03–3.86), upadacitinib 15 mg (RR = 5.30, 95% CI = 4.19–6.71) and upadacitinib 30 mg (RR = 7.31, 95% CI = 5.81–9.21)] showed a statistically significantly higher rate of achieving an IGA response of 0 or 1 compared to patients in the placebo group. Abrocitinib 200 mg (*I* ^2^ = 64.4%, *p* = 0.024) showed significant heterogeneity. No significant heterogeneity was observed for the other groups [abrocitinib 100 mg (*I* ^2^ = 31.2%, *p* = 0.213), baricitinib 1 mg (*I* ^2^ = 0.0%, *p* = 0.910), baricitinib 2 mg (*I* ^2^ = 30.4%, *p* = 0.219), baricitinib 4 mg (*I* ^2^ = 0.0%, *p* = 0.589), upadacitinib 15 mg (*I* ^2^ = 60.9%, *p* = 0.053) and upadacitinib 30 mg (*I* ^2^ = 54.0%, *p* = 0.089)].

FIGURE 5Forest plots for the proportion of patients achieving an IGA of 0 or 1 on oral abrocitinib 100 mg (a), oral abrocitinib 200 mg (b), oral baricitinib 1 mg (c), oral baricitinib 2 mg (d), oral baricitinib 4 mg (e), oral upadacitinib 15 mg (f), and oral upadacitinib 30 mg (g) compared to patients on placebo

**Figure:**
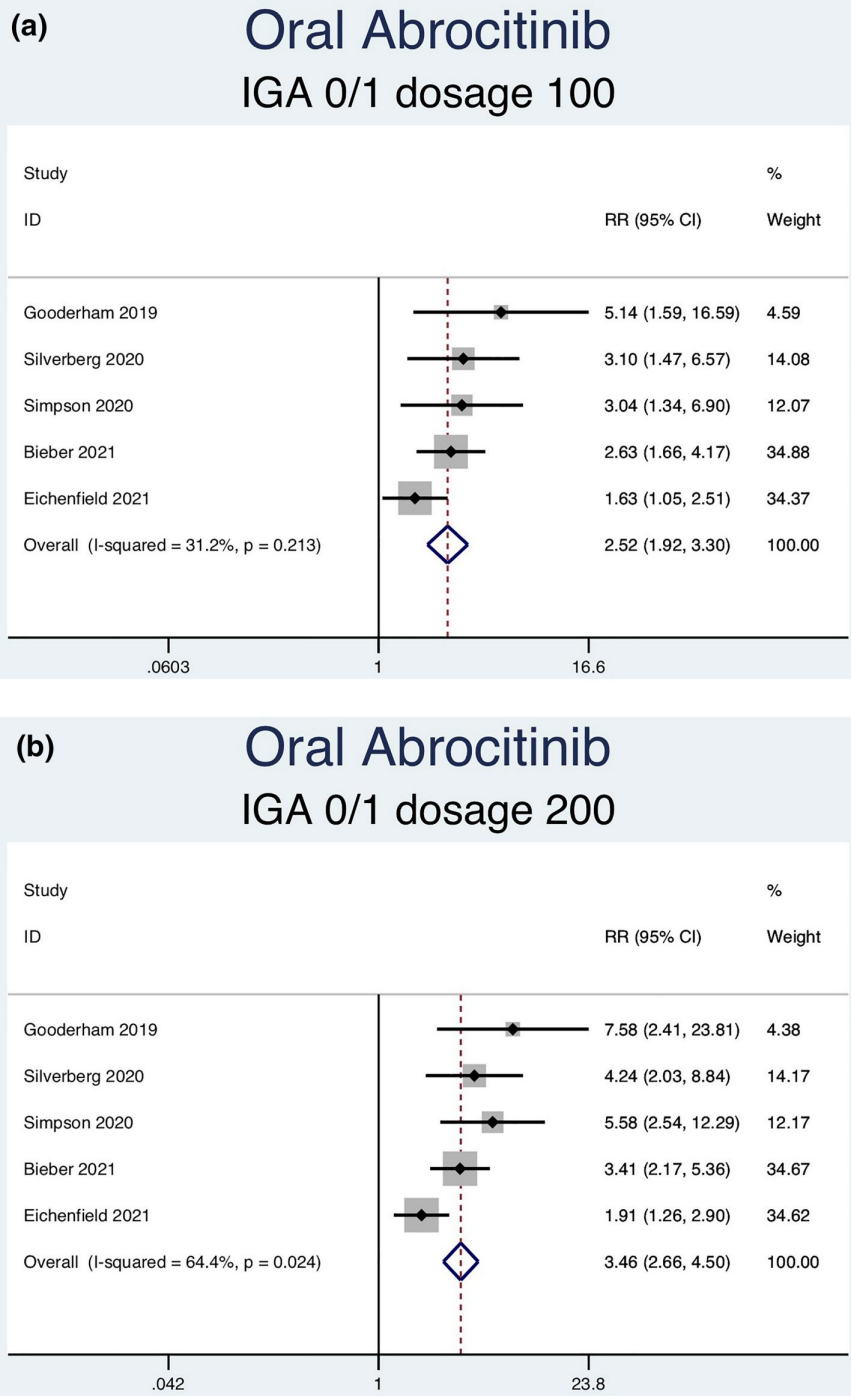

**Figure:**
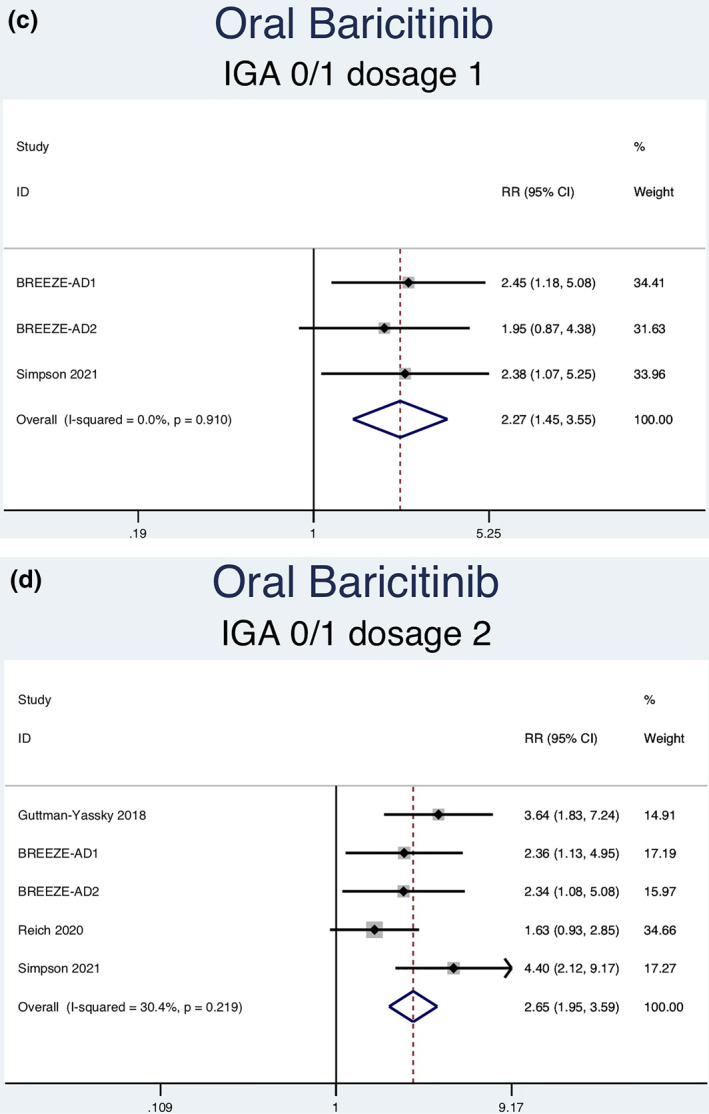

**Figure:**
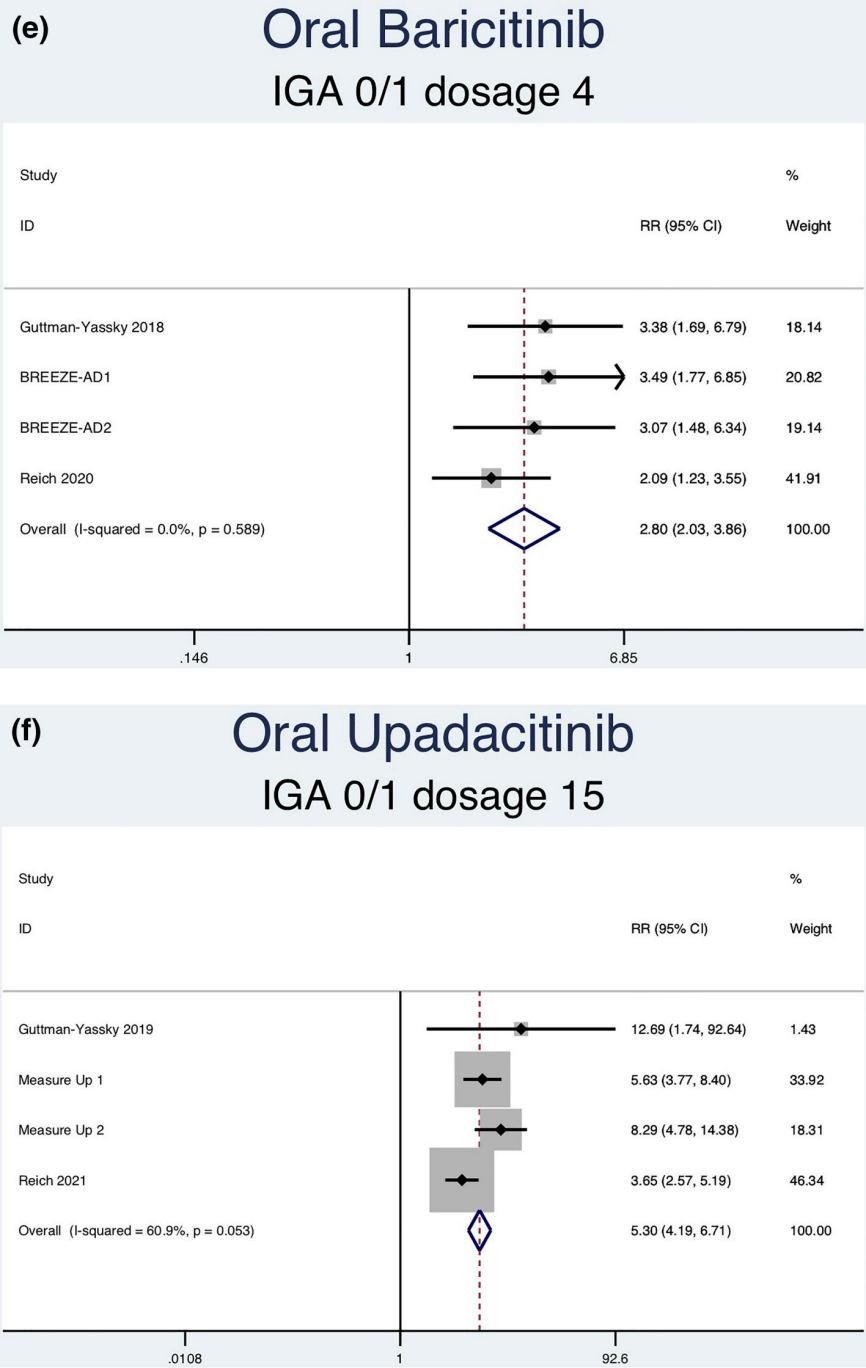

**Figure:**
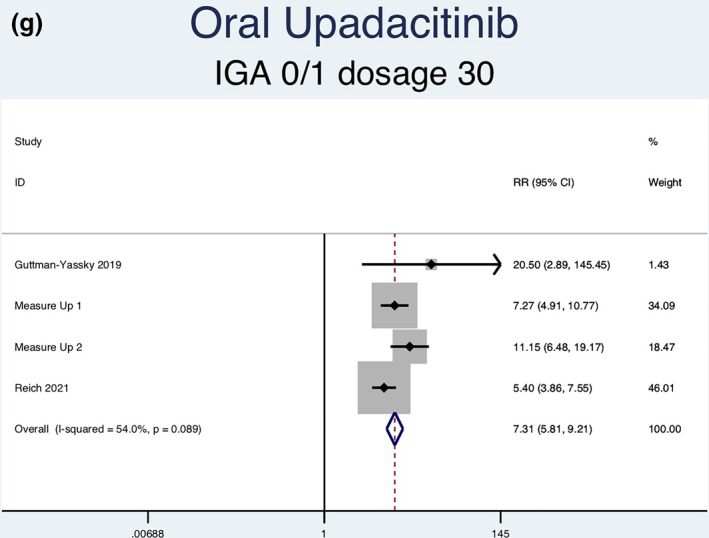

Thirteen RCTs (6927 patients) reporting a greater than or equal to 4‐point improvement in pruritus NRS as an outcome were analysed. Seven separate meta‐analyses (Figure 6a–g) were performed for patients on abrocitinib 100 mg, abrocitinib 200 mg, baricitinib 1 mg, baricitinib 2 mg, baricitinib 4 mg, upadacitinib 15 mg and upadacitinib 30 mg. All groups [abrocitinib 100 mg (RR = 2.02, 95% CI = 1.65–2.48), abrocitinib 200 mg (RR = 2.63, 95% CI = 2.15–3.20), baricitinib 1 mg (RR = 1.74, 95% CI = 1.09–2.79), baricitinib 2 mg (RR = 2.35, 95% CI = 1.72–3.22), baricitinib 4 mg (RR = 2.61, 95% CI = 1.90–3.60), upadacitinib 15 mg (RR = 4.14, 95% CI = 3.41–5.03) and upadacitinib 30 mg (RR = 5.24, 95% CI = 4.34–6.34)] showed a statistically significantly higher rate of achieving ≥ 4‐point improvement in pruritus NRS compared to placebo. Significant heterogeneity was seen for abrocitinib 200 mg (*I* ^2^ = 63.1%, *p* = 0.029). No significant heterogeneity was observed for any other group [abrocitinib 100 mg (*I* ^2^ = 45.1%, *p* = 0.121), baricitinib 1 mg (*I* ^2^ = 20.3%, *p* = 0.285), baricitinib 2 mg (*I* ^2^ = 52.0%, *p* = 0.100), baricitinib 4 mg (*I* ^2^ = 18.4%, *p* = 0.294), upadacitinib 15 mg (*I* ^2^ = 7.4%, *p* = 0.356) and upadacitinib 30 mg (*I* ^2^ = 19.1%, *p* = 0.295)].

FIGURE 6Forest plots for the patients achieving a greater than or equal to 4‐point improvement in pruritus NRS on oral abrocitinib 100 mg (a), oral abrocitinib 200 mg (b), oral baricitinib 1 mg (c), oral baricitinib 2 mg (d), oral baricitinib 4 mg (e), oral upadacitinib 15 mg (f), and oral upadacitinib 30 mg (g) compared to patients on placebo

**Figure:**
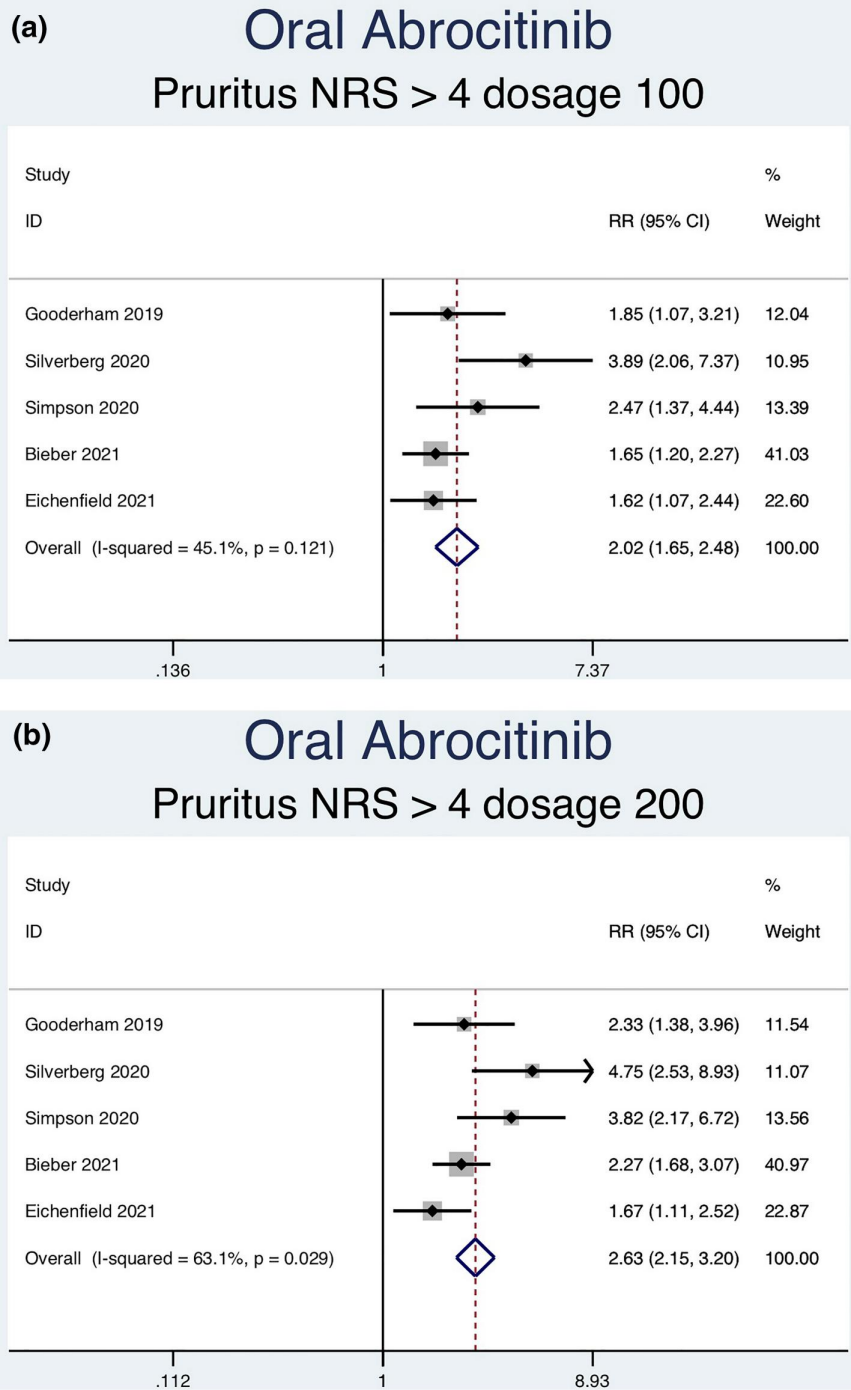

**Figure:**
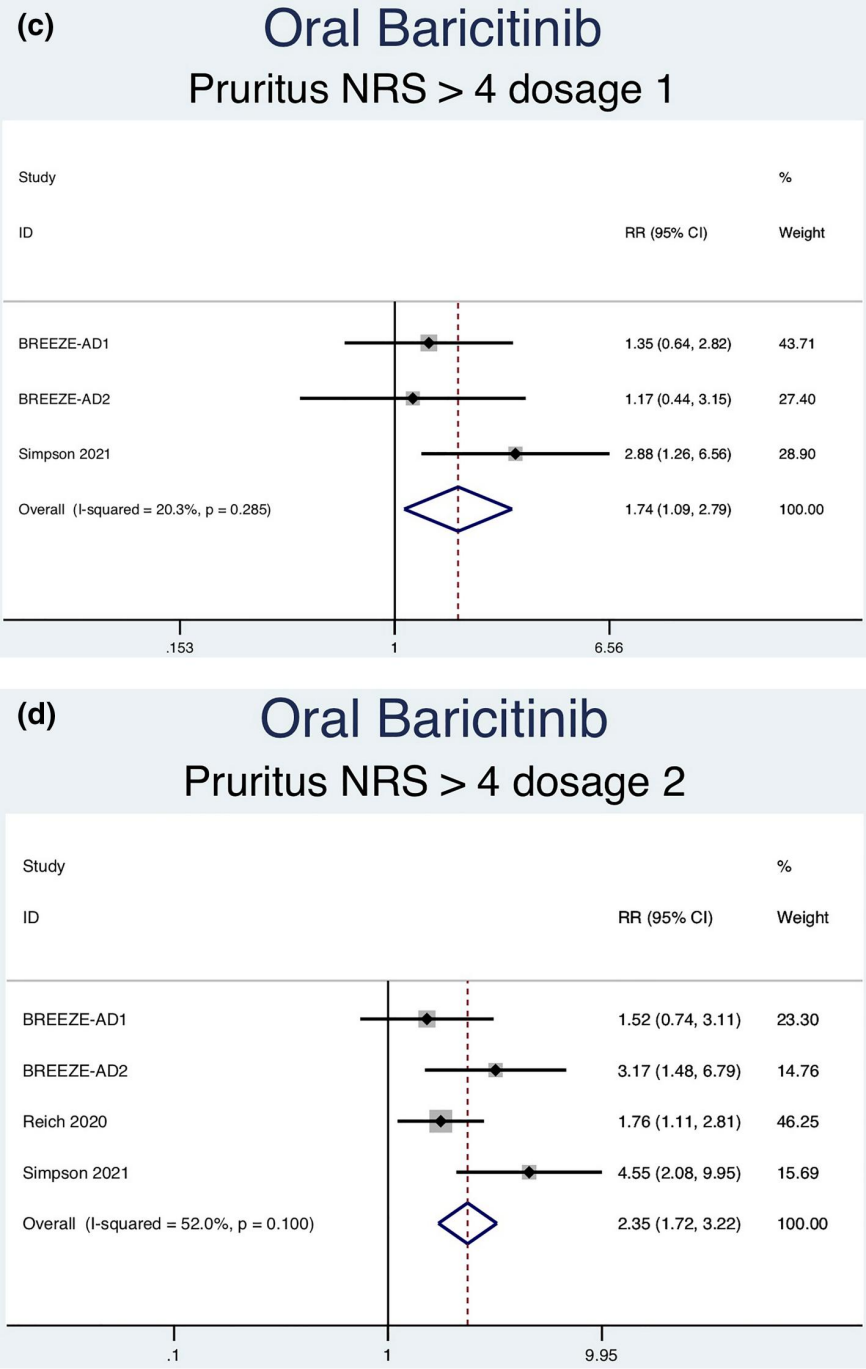

**Figure:**
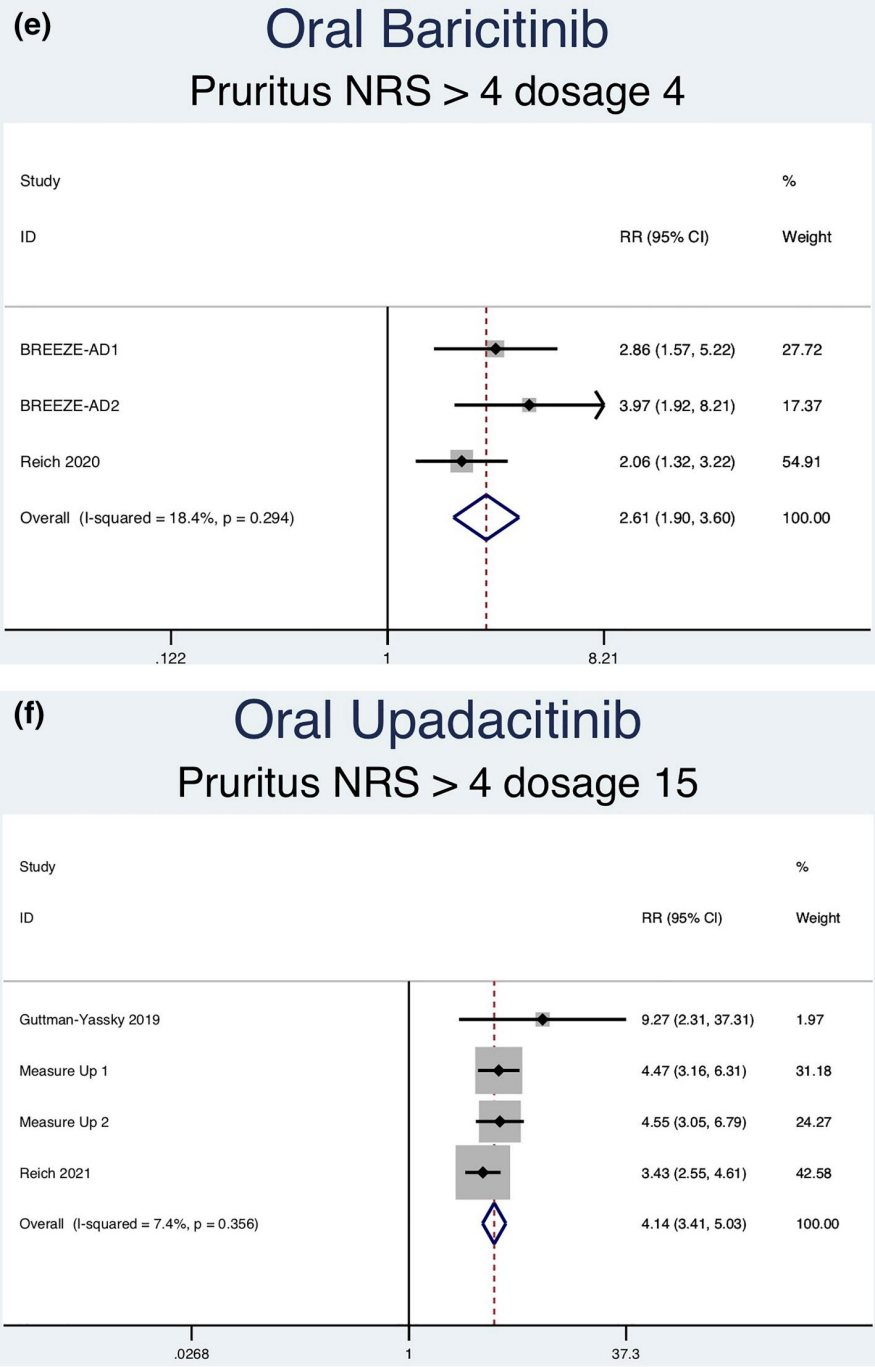

**Figure:**
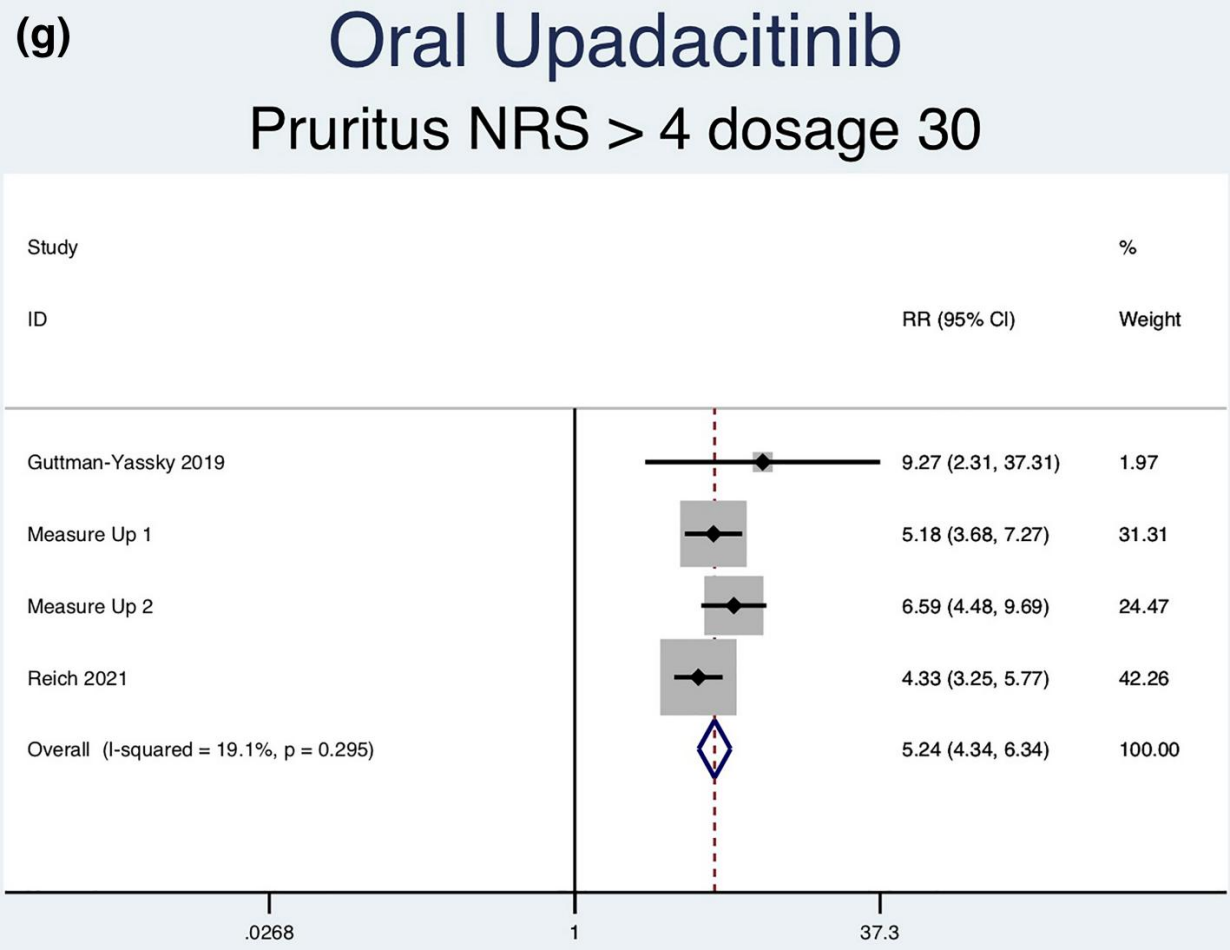

### 4.5 Safety outcomes

Of note, there were three subjects that developed a thromboembolic event. One each in the placebo, abrocitinib 200 mg and baricitinib 4 mg groups. There was one death in the abrocitinib 100 mg group. A full summary of adverse events for the placebo and treatment groups are shown in Table 4.

**TABLE 4:** Summary of adverse events

Drug name/ Timepoint (weeks)	Treatment arm	Nausea *n* (%)	Headache *n* (%)	All infections *n* (%)	Serious infections *n* (%)	Herpes zoster *n* (%)	Elevated CPK *n* (%)	Thrombo‐cytopenia *n* (%)	Malignancy *n* (%)	Thrombo‐embolism *n* (%)	Death *n* (%)
Abrocitinib (12 weeks)	Placebo	8 (1.8)	19 (4.4)	82 (18.9)	2 (0.5)	0	3 (1.0)	1 (0.3)	0	0	0
	100 mg	44 (6.3)	41 (5.8)	177 (25.2)	8 (1.1)	4 (0.6)	7 (1.4)	0	0	0	1 (0.1)
	200 mg	103 (15.2)	54 (8.0)	141 (20.8)	2 (0.3)	8 (1.3)	10 (2.1)	8 (1.5)	0	1 (0.1)	0
Baricitinib (16 weeks)	Placebo	2 (0.7)	23 (3.3)	131 (16.4)	5 (0.6)	1 (0.3)	4 (0.6)	0	2 (0.3)	0	0
	1mg	1 (0.8)	18 (4.5)	66 (16.5)	4 (1.0)	1 (0.7)	5 (2.0)	0	0	0	0
	2mg	3 (1.9)	28 (6.5)	102 (19.0)	1 (0.2)	2 (0.7)	6 (1.5)	0	0	0	0
	4mg	1 (0.6)	26 (9.1)	87 (21.9)	1 (0.3)	0	16 (4.0)	0	0	1 (0.3)	0
Upadacitinib (16 weeks)	Placebo	1 (2.4)	39 (4.3)	124 (13.7)	5 (0.6)	5 (0.6)	21 (2.3)	0	0	1 (0.1)	0
	15 mg	1 (2.4)	50 (5.6)	149 (16.6)	7 (0.8)	14 (1.6)	41 (4.6)	0	3 (0.3)	0	0
	30 mg	3 (7.1)	57 (6.3)	188 (20.8)	4 (0.4)	14 (1.5)	50 (5.5)	1 (0.3)	5 (0.6)	0	0

Abbreviations: %, percent of patients; CPK, creatinine phosphokinase; n, number of patients.

### 4.6 Publication bias

The test of publication bias was not performed.

## 5 DISCUSSION

In our systematic review and meta‐analysis, we found that all three JAK inhibitors were more effective than placebo in achieving EASI‐75, percent change in EASI score, an IGA response of 0 or 1, and a greater than or equal to 4‐point improvement in pruritus NRS in patients with AD. Overall, the majority of endpoints showed no significant heterogeneity for each dose. Our results are similar to prior studies.^27^, ^28^ One strength of our analysis is that by performing meta‐analyses on specific drugs and their dosages, we attempted to provide better comparisons among the different JAK inhibitors at varying dosages.

We found upadacitinib to have the greatest efficacy out of the three JAK inhibitors analysed. Upadacitinib 30 mg was statistically significantly more effective than all the other doses of JAK inhibitors besides abrocitinib 200 mg and upadacitinib 15 mg in achieving EASI‐75 response compared to placebo. Upadacitinib 30 mg also had a significant decrease in percent EASI compared to every dose of the other JAK inhibitors and upadacitinib 15 mg. Abrocitinib 200 mg had a statistically significant decrease in percent EASI compared to each of the three doses of baricitinib. Additionally, upadacitinib 30 mg had a statistically significantly higher rate of achieving an IGA response of 0 or 1 compared to every dose of abrocitinib and baricitinib. However, the difference between upadacitinib 30 mg and upadacitinib 15 mg in IGA 0/1 response was not statistically significant. Finally, upadacitinib 30 mg had a statistically significantly higher rate of achieving a greater than or equal to 4‐point improvement in pruritus NRS compared to every dose of abrocitinib and baricitinib. Upadacitinib 15 mg was also found to be significantly more efficacious in three outcomes (% change in EASI, IGA 0/1 and ≥ 4‐point improvement in pruritus NRS) except for abrocitinib 200 mg (% change in EASI and IGA 0/1) and baricitinib 4 mg (≥ 4‐point improvement in pruritus NRS). Based on these results, upadacitinib at a dose of 30 mg appears to be the most efficacious oral treatment of the three JAK inhibitors examined for moderate‐to‐severe AD.

Another strength of our study is that we attempted to use the highest level of data available for our analyses. Data were only included if the results came from RCTs and if there were multiple RCTs for the specific JAK inhibitor and dose.

A limitation of our study was the lack of available trials. Several JAK inhibitors only had one RCT with results published. These were not included in our meta‐analysis. Another limitation was the short duration of follow‐up included in the trials. Furthermore, the safety data was also limited, and a meta‐analysis could not be performed on the adverse effects.

JAK inhibitors have been touted to have acceptable safety profile in multiple studies.^18^, ^20^, ^24^ In our study, there was a higher percentage of patients with herpes zoster in the treatment group compared to placebo. This finding is consistent with a prior meta‐analysis that found an increase in herpes zoster incidence in rheumatoid arthritis population taking JAK inhibitors.^29^ Furthermore, the immunomodulatory properties of JAK inhibitors lead to concerns of increased malignancy. In our study, eight patients in the upadacitinib group and two patients in the placebo group had an adverse event of malignancy. In the upadacitinib group, there were five patients with non‐melanoma skin cancer and one patient each with anal, gastric and breast cancer. All the cancers, except for two of the non‐melanoma skin cancers, were considered not related to drug. However, long‐term safety data would be required to further evaluate the risk of malignancy.

Another area of concern with JAK inhibitors is whether they cause a predisposition to venous thromboembolism (VTE). Two recent studies evaluated the safety of JAK inhibitors and both studies concluded that there was no significant increase in the occurrence of VTE in patients with rheumatoid arthritis, inflammatory bowel disease, or other immune‐mediated diseases.^30^, ^31^ Neither study included AD subjects. However, the Food and Drug Administration has restricted the use of baricitinib to 2 mg daily due to concerns of increased VTE risk. Furthermore, another study in rheumatoid arthritis patients greater than 50 years‐old with at least one cardiovascular risk factor found an increased risk for VTE in the tofacitinib 10 mg twice daily group during interim analysis. As a result, all the subjects were switched to tofacitinib 5 mg twice a day.^32^ In our study, one patient in each of the placebo, abrocitinib, and baricitinib groups developed a thromboembolic event. In order to further study an event as rare as VTE, evaluation of a larger patient population or registered database is required.

In conclusion, our study demonstrated that JAK inhibitors are an effective treatment for AD. We found that upadacitinib, particularly at 30 mg, was significantly more efficacious than both abrocitinib and baricitinib in every outcome analysed. Clinical trials with comparisons among the JAK inhibitors will be needed to confirm these results. More studies will also be needed to explore the long‐term efficacy and safety of these molecules.

## AUTHOR CONTRIBUTIONS

**Kevin P. Lee:** Conceptualisation (equal); Data curation (equal); Investigation (equal); Methodology (equal); Writing–original draft (lead); Writing–review and editing (lead). **John Plante:** Data curation (equal); Formal analysis (equal); Investigation (equal); Methodology (equal); Resources (equal); Writing–original draft (supporting); Writing–review and editing (supporting). **Jeffrey E. Korte:** Data curation (lead); Formal analysis (lead); Methodology (equal); Resources (equal); Software (lead). **Dirk M. Elston:** Conceptualisation (lead); Data curation (equal); Investigation (equal); Methodology (lead); Project administration (lead); Resources (lead); Writing–original draft (equal); Writing–review and editing (equal).

## CONFLICT OF INTEREST

The authors declare no conflict of interest.

## Data Availability

The data that support the findings of this study are available from the corresponding author upon reasonable request.
